# Grading of endometrial cancer using ^1^H HR-MAS NMR-based metabolomics

**DOI:** 10.1038/s41598-021-97505-y

**Published:** 2021-09-13

**Authors:** Agnieszka Skorupa, Michał Poński, Mateusz Ciszek, Bartosz Cichoń, Mateusz Klimek, Andrzej Witek, Sławomir Pakuło, Łukasz Boguszewicz, Maria Sokół

**Affiliations:** 1 Department of Medical Physics, Maria Skłodowska-Curie National Research Institute of Oncology, Gliwice Branch, 44-102 Gliwice, Poland; 2 Department of Gynaecology and Obstetrics, Medical University of Silesia in Katowice, Medyków 14, 40-752 Katowice, Poland; 3 Tumor Pathology Department, Maria Skłodowska-Curie National Research Institute of Oncology, Gliwice Branch, 44-102 Gliwice, Poland

**Keywords:** Cancer metabolism, Gynaecological cancer, Tumour biomarkers, Metabolomics, Metabolomics

## Abstract

The tissue metabolomic characteristics associated with endometrial cancer (EC) at different grades were studied using high resolution (400 MHz) magic angle spinning (HR-MAS) proton spectroscopy. The metabolic profiles were obtained from 64 patients (14 with grade 1 (G1), 33 with grade 2 (G2) and 17 with grade 3 (G3) tumors) and compared with the profile acquired from 10 patients with the benign disorders. OPLS-DA revealed increased valine, isoleucine, leucine, hypotaurine, serine, lysine, ethanolamine, choline and decreased creatine, creatinine, glutathione, ascorbate, glutamate, phosphoethanolamine and scyllo-inositol in all EC grades in reference to the non-transformed tissue. The increased levels of taurine was additionally detected in the G1 and G2 tumors in comparison to the control tissue, while the elevated glycine, *N*-acetyl compound and lactate—in the G1 and G3 tumors. The metabolic features typical for the G1 tumors are the increased dimethyl sulfone, phosphocholine, and decreased glycerophosphocholine and glutamine levels, while the decreased myo-inositol level is characteristic for the G2 and G3 tumors. The elevated 3-hydroxybutyrate, alanine and betaine levels were observed in the G3 tumors. The differences between the grade G1 and G3 malignances were mainly related to the perturbations of phosphoethanolamine and phosphocholine biosynthesis, inositol, betaine, serine and glycine metabolism. The statistical significance of the OPLS-DA modeling was also verified by an univariate analysis. HR-MAS NMR based metabolomics provides an useful insight into the metabolic reprogramming in endometrial cancer.

## Introduction

Endometrial cancers, ECs, are the most common cancers of the genital organ in women^1^, being frequently associated with various metabolic disorders, including obesity, hypertension and diabetes, known as the metabolic triad^2^. Their traditional classification is based either on the clinical and endocrine features (e.g. the types I and II) or on the histopathological characteristics (e.g. endometrioid, serous, or clear-cell adenocarcinoma). Type I, more common (~ 70 to 80%) and of a lower risk, consists of estrogen-driven endometrioid, low grade, diploid, hormone receptor-positive tumors, whereas type II comprises less common (20–30%), more aggressive, nonestrogen-driven cancers of a non-endometrioid histology, high-grade, aneuploid, poorly differentiated, hormone receptor negative and associated with a higher risk of metastasis and a poor prognosis^3–5^. According to current practice, ECs are assigned the FIGO (International Federation of Gynaecology and Obstetrics) grades based on the degree of glandular differentiation^6,7^, however, in one-third or more cases the pathologic accuracy of the tumor grade (mainly in grade 3 endometrioid and serous cancers) is occasionally low due to a poor diagnostic reproducibility^2^.

Thus, although this dualistic classification system remains the gold standard, a new classification by The Cancer Genome Atlas (TCGA) Consortium, based on the molecular features, is starting to be integrated into a clinical practice^8^. Because of a substantial inter-tumor heterogeneity within the EC tumor types, various subtypes have been identified mainly by the gene expression profiles^9–13^. Genomic profiling informs of what will happen in the tumor, but it is metabolomics that delivers the objective summary information on all current genotypic, environmental and physiological effects^14^ that mirror the biological features as well as the clinical behaviors of individual tumors.

Metabolomic studies have already confirmed that cancer is a metabolic disorder with profound alterations of the critical pathways, such as glycolysis, tricarboxylic acid cycle, choline and fatty acid metabolism in cancer cells^15^, typical for neoplastic transformation, cellular proliferation and inflammatory response to malignancy^16^. Also in EC the biomarkers of the mentioned processes are useful for cancer detection or treatment monitoring and can be linked to specific histological or molecular subtypes of EC, thus, to explore also the intra-tumor heterogeneity^16–19^, and to direct the targeted treatments or to predict the EC outcome^13,20^.

Nuclear magnetic resonance (NMR) spectroscopy is an essential analytical technique in metabolomics and in the studies of metabolic rearrangements subsequent to malignant transformation. NMR-based metabolomics can be easily applied to analyze different kinds of human samples, including blood plasma/serum^21^. Since endometrial tissue communicates with the blood directly or through the extracellular fluids, several metabolites may be secreted into the blood stream^22^. However, the diagnostic potential of these blood-based metabolites is claimed to be limited in EC by dilution, especially in case of small and early stage tumors^13^. In spite of this difficulty, the serum NMR metabolomic signature of endometrial cancer was found to differ from that of the healthy controls and from that of benign endometrial disease as well as from other gynecological cancers^23^. Another NMR approach, using high resolution magic angle spinning (HR-MAS) proton (1H) spectroscopy, is free from the systemic metabolic effects, thus provides a detailed information on the tissue metabolic profile within the tumor site and identifies the cancer characteristics that can have a clinical impact. HR-MAS NMR has a high degree of reproducibility and a non-destructive nature, which allows the specimens to be evaluated by microscopy after the spectral analysis, making direct comparisons to morphologic characteristics feasible^24^. Despite these advantages, there are still few applications of this method in the studies of the EC metabolomic phenotypes. Recently, Trousil et al.^25^ have identified the metabolic patterns that illustrate a severely altered phenotype in EC. They involved the proliferation-associated markers (e.g. various amino acids and the nucleobase uracil, which are indicative of high transcriptional and proliferative activity), maintenance of intracellular redox homeostasis (glutathione), osmoregulation (inositols, taurine) and glucose.

In this study, we used HR-MAS NMR to define the tissue metabolomic characteristics associated with EC at different grades and to explore the potentials of these molecular phenotypic profiles for the diagnosis of endometrial cancers.

## Materials and methods

### Patients

The study was approved by the Bioethics Committee of the Medical University of Silesia in Katowice (protocol code KNW/022/KB1/118/17) and informed consent was obtained from all patients. All research was performed in accordance with relevant guidelines and regulations.

The study included 64 postmenopausal women hospitalized in 2017–2019 at the Department of Gynecology and Obstetrics of the Silesian Medical University in Katowice with diagnosed endometrial cancer confirmed by histopathological examination. The control group consists of 10 postmenopausal patients hospitalized in the clinic due to prolapse of the reproductive organ, leiomyomas, cystadenomas. The clinical characteristics of the participants of the study (cancer grade, cancer stage, BMI, age, time from the menopause and the occurrence of the most common comorbidities—diabetes mellitus and hypertension) are shown in Table 1. The patients receiving the hormone replacement therapy were excluded from the studied group. The patients were divided according to the degree of a pathomorphological maturity (proportion of glandular differentiation) into 3 groups: G1 (≥ 95% gland formation, 14 patients), G2 (= 95–50% gland formation, 34 patients), G3 (< 50% gland formation, 17 patients). The Kruskal–Wallis test followed by multiple comparisons of the mean ranks indicates that these groups are matched on age and time from the menopause (p < 0.05). As far as BMI is concerned, the difference between the G1 and G2 tumors is at the border of a statistical significance (p = 0.0464). Importantly, the groups of the patients diagnosed with the tumors of different grades are not matched according to the disease stage. Specifically, all patients with the G1 tumors were diagnosed with stage 1, while those with the G3 tumors with stages 2–3. 64% of the patients with the G2 tumors were diagnosed with stage 1, while the rest of them (36%) with stages 2–3.

**Table 1:** Clinical characteristics of the patients.

Grade	Number of patients	Disease stage	Patients/stage	Age (years) Mean ± SD	Time from menopause (years) Mean ± SD	BMI (kg/m^2^) Mean ± SD	Diabetes mellitus and/or hypertension
G1	14	1	14	64.86 ± 9.12	13.66 ± 7.08	26.85 ± 1.99	57% (8/14)
G2	33	1	21	70.24 ± 7.39	17.15 ± 9.23	30.51 ± 5.62	57% (19/33)
		2	7				
		3	5				
G3	17	2	8	68 ± 8.39	14.91 ± 7.45	29.43 ± 3.25	47% (8/17)
		3	9				
Control tissue	10	–	–	62 ± 7.32	10.22 ± 6.04	27.03 ± 2.17	–

The uterus was dissected in the frontal line from the cervical canal to its bottom to visualize the uterine cavity. The tissue samples were collected in the operating room, immediately after the uterus was removed, following the sterility rules. Then the samples were transported in a dewar vessel to the molecular laboratory and stored in a freezer at − 80 °C until they were subjected to the NMR analyzes.

### HR MAS NMR

HR MAS NMR experiments were performed on a Bruker Avance III 400 MHz spectrometer operating at a proton frequency of 400.17 MHz equipped with ^1^H optimized 4-mm ^1^H/^13^C MAS probe. Before experiments the frozen tissue samples were cut to fit 30 µl disposable Kel-F inserts filled with a 5 µl cold solution of 25 mM sodium formate in D20 for shimming and locking purposes. The inserts were placed in the zirconium HR-MAS rotors (diameter of 4 mm). The NMR measurements were performed at 4 °C with a magic angle spinning at 4000 Hz. The water presaturated 1D proton Carr-Purcell-Meiboom-Gill (CPMG; sequence: cpmgpr1d, Bruker) spectra were acquired using the following parameters: a relaxation delay of 4 s, an effective echo time of 240 ms, a spectral width of 20 ppm, 65,536 points, an acquisition time of 4.08 s and 256 scans. The metabolite signals were assigned based on 2D NMR spectra (J-resolved, 1H–1H TOCSY and 1H–13C HSQC) acquired for selected samples and using literature data^25^. The quality control procedures are presented in the Supplementary material of the paper (Quality Control).

### Data analysis

**Figure 1:**
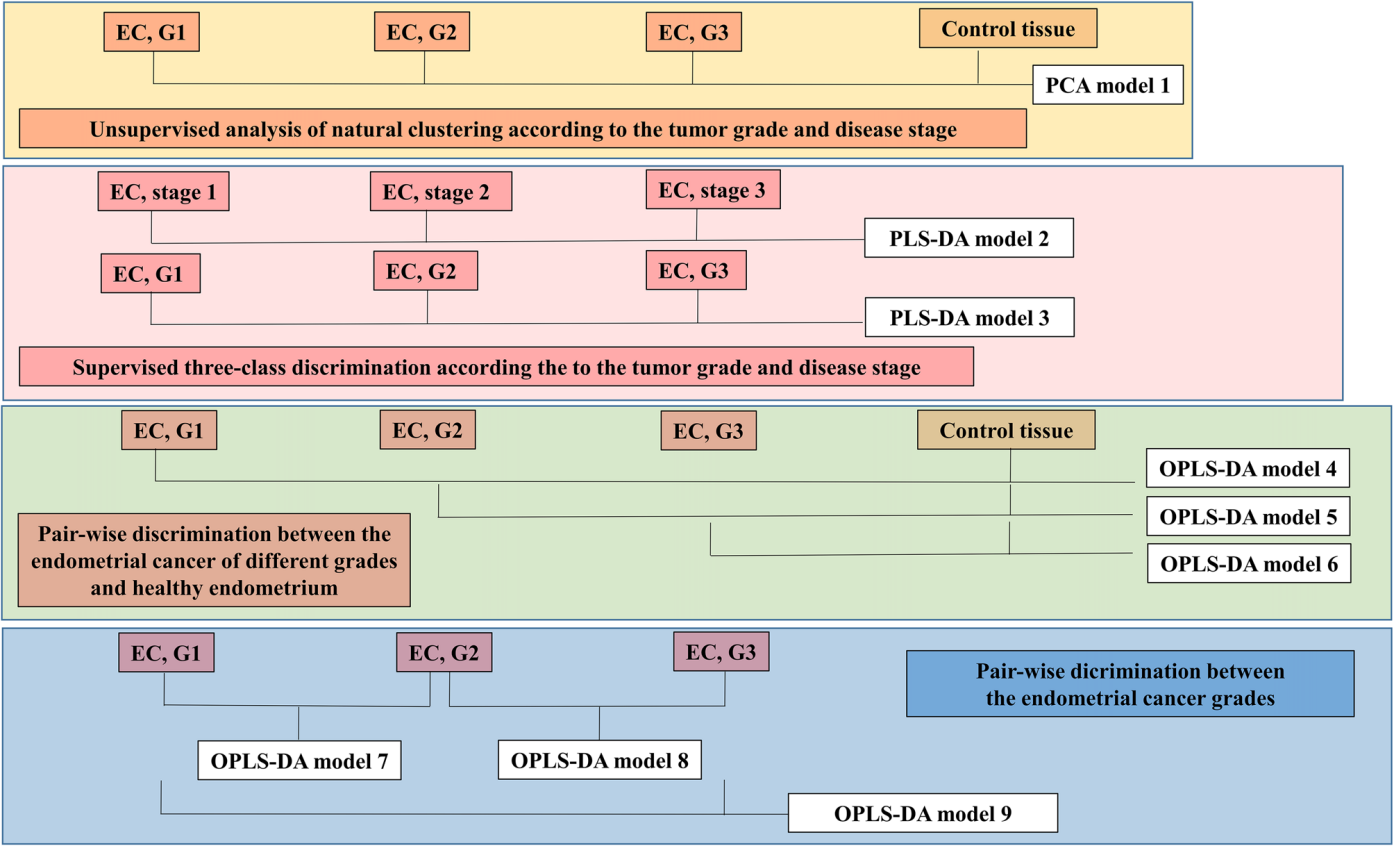
The scheme presenting the constructed multivariate models.

The initial preprocessing of the HR-MAS NMR CPMG spectra was performed in TopSpin 3.1 software (Bruker BioSpin GmbH). The free induction decay signals were multiplied by an exponential function (0.3 Hz), Fourier-transformed, phased and baseline corrected. The spectra were referenced to formate peak (at 8.44 ppm) in Mestrenova software (Santiago de Compostela, Spain). Then, the fine peak alignment was conducted using the FFT/peak matching method in SpecAlign 2.4 software (Oxford University)^26^. The spectral region from 0.8 to 4.8 ppm was normalized using the probabilistic quotient normalization technique. Median spectrum from control tissue served as a reference spectrum in this procedure. The pre-processed data were imported to SIMCA-P 15.0 software (Umetrics, Sweden) for multivariate modeling. The spectra were analyzed at full resolution. Before the modeling, the data were mean centered and Pareto scaled. The scheme of the analyses is presented in Fig. 1.

Principal component analysis (PCA) was used to obtain the initial information about the natural grouping of the spectra acquired from the cancer samples (according to the tumor grade and the disease stage) and the healthy tissue (model 1). The clustering of tumors according to grade and stage was also examined using partial least-squares discriminant analysis (PLS-DA) (models 2 and 3). The metabolic differences between the tumors of different grades and the non-transformed endometrium were examined using the supervised Orthogonal partial least squares discriminant analysis (OPLS-DA) (models 4–6). OPLS-DA was also exploited to discriminate between the endometrial cancer grades (models 7–9).

The results were interpreted by means of the scores and loadings plots. The combination of the VIP (Variable Importance at Projection) scores and the loadings scaled as the correlation coefficients [the p(corr) values] was used for identification of the potential biomarkers. The VIP values > 0.7 and |p(corr)[1]|> 0.45 were considered as the inclusion criteria for selection of the discriminating metabolites. The two class OPLS-DA models [G1 vs. G2 (model 7) and G2 vs. G3 (model 8)] were compared to identify the common and unique metabolic features of the G1 and G3 tumors in reference to the intermediate G2 grade tumors. The interpretation was based on the position of the variables in the SUS plot. The metabolites located along the diagonals represent the shared features, while the off-diagonal ones are unique for one of the models^27^.

The predictive performance of the supervised OPLS-DA models was evaluated using seven-fold cross-validation. Each model was characterized by the fraction of the explained variation (R2) and the fraction of the predicted variation (Q2). The statistical significance of the developed models was also assessed using the permutation testing (100 permutations) and cross validation analysis of variance (CV-Anova).

To evaluate classification accuracy of multivariate models differentiating G1 vs. G2, G2 vs. G3 and G1 vs. G3 tumors the predicted Y-scores computed from cross validation were subjected to ROC (receiver operating characteristic) curves analysis in Metaboanalyst 4.0 software^28^. This analysis was also performed for the individual metabolite levels.

The metabolic differences between the cancer and control tissues and between the different grades were also analyzed using univariate statistics. The signal integrals corresponding to the specific metabolites were determined by a direct integration using Mestrenova 10.0 software (Mestrelab Research). To obtain more accurate information about the levels of the choline containing compounds (phosphocholine and glycerophosphocholine), the spectral region from 3.18 to 3.32 ppm was additionally line fitted using automatic deconvolution analysis. The signal integrals were subjected to the Kruskal–Wallis test followed by multiple comparisons of the mean ranks. The changes were considered statistically significant for the p values < 0.05.

Although the direct analysis of the influence of the disease advancement on the metabolic profiles within each pathomorphological grade group is difficult, several multivariate models were constructed to shed some light on this problem (Supplementary materials, Figure S3). Two separate OPLS-DA models distinguishing the G2 (stage 1) tumors and the G2 (stage 2 + 3) tumors from the control tissue (OPLS-DA models S1 and S2, respectively) were compared to each other by means of the SUS (Shared and Unique Structures) plot—it is constructed by combining the p(corr) values obtained for the predictive components from the two abovementioned models. To examine the confounding effect of the disease stage on the metabolic differences between G1 and G2 endometrial cancer, the OPLS-DA model S3 was built based on the G1 and G2 (stage 1) tumors. The patients with the G2 and G3 tumors with more advanced disease stage (stages 2 + 3) were included in the development of the OPLS-DA model S4.

Preliminary multivariate analysis was conducted for the merged aliphatic (0.8–4.8 ppm, apodized with an exponential function 0.3 Hz) and aromatic (5.2–8.4 ppm, apodized with an exponential function 3 Hz) regions of the HR MAS NMR spectra. These results are presented in Supplementary Materials [multivariate analysis of the merged aliphatic (0.8–4.8 ppm) and aromatic (5.2–8.4 ppm) regions of the HR MAS NMR spectra]. However, due to a markedly lower signal intensity of the metabolites resonating in the aromatic region, their contribution to the model was not sufficient to consider them as potential biomarkers according to the criteria accepted in our work. Therefore, separate multivariate analysis of the aromatic region was conducted. The results are also shown in the Supplementary Materials (Multivariate analysis of the spectral region from 5.2 to 8.4 ppm).

### Metabolic pathways enrichment and impact analysis

The signal integrals corresponding to the specific metabolites were subjected to the pathway impact and enrichment analysis using MetaboAnalyst 4.0 platform with the Small Molecule Pathway Database (SMPDB)—a database containing more than 350 small-molecule pathways found in humans, designed to facilitate clinical ‘omics’ studies, with a specific emphasis on clinical metabolomics^28,29^. The pathways were considered important when the pathway impacts were higher than the threshold set to be 0.1 and when the Holm p values obtained from the enrichment analysis were below 0.05.

## Results

**Figure 2:**
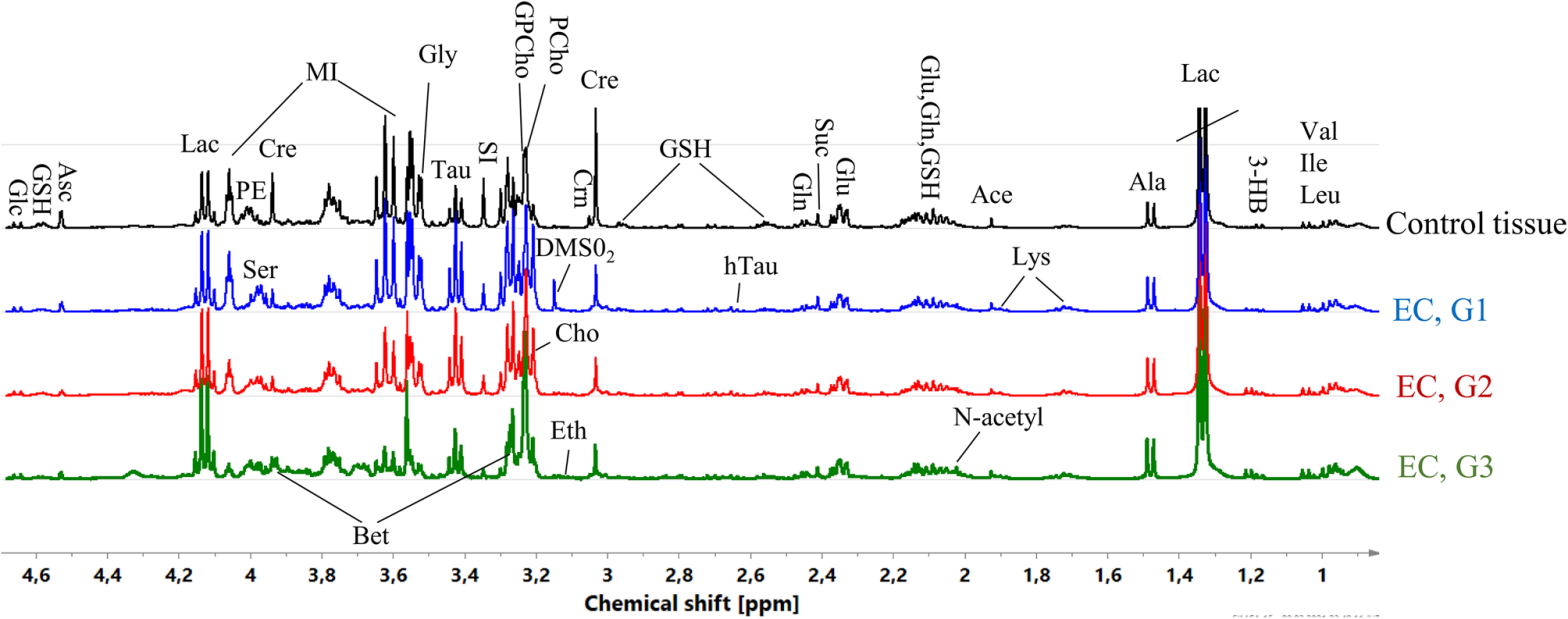
Average ^1^H HR-MAS NMR CPMG spectra obtained from the G1, G2 and G3 endometrial tumors and the healthy endometrium. The assigned signals: *Val* valine, *Ile* isoleucine, *Leu* leucine, *3-HB* 3-hydroxybutyrate, *Lac* lactate, *Ala* alanine, *Ace* acetate, *Glu* glutamate, *Gln* glutamine, *GSH* glutathione, *Lys* lysine, *N-acetyl* *N*-acetyl compound, *hTau* hypotaurine, *Suc* succinate, *Cre* creatine, *Crn* creatinine, *PCho* phosphocholine, *GPCho* glycerophosphocholine, *Cho* choline, *DMSO*_*2*_ dimethyl sulfone, *Eth* ethanolamine, *SI* scyllo-inositol, *Tau* taurine, *Gly* glycine, *MI* myo-inositol, *PE* phoshoethanolamine, *Ser* serine, *Bet* betaine, *Asc* ascorbate, *Glc* glucose. The image was created using SIMCA-P 15.0 software package (https://www.sartorius.com).

Figure 2 presents the average CPMG spectra acquired from the different grades of EC and the healthy endometrium. The assignments of NMR signals are presented in Table S1 (Supplementary materials).

### Unsupervised analysis of natural clustering of patients according to the tumor grade and disease stage

**Figure 3:**
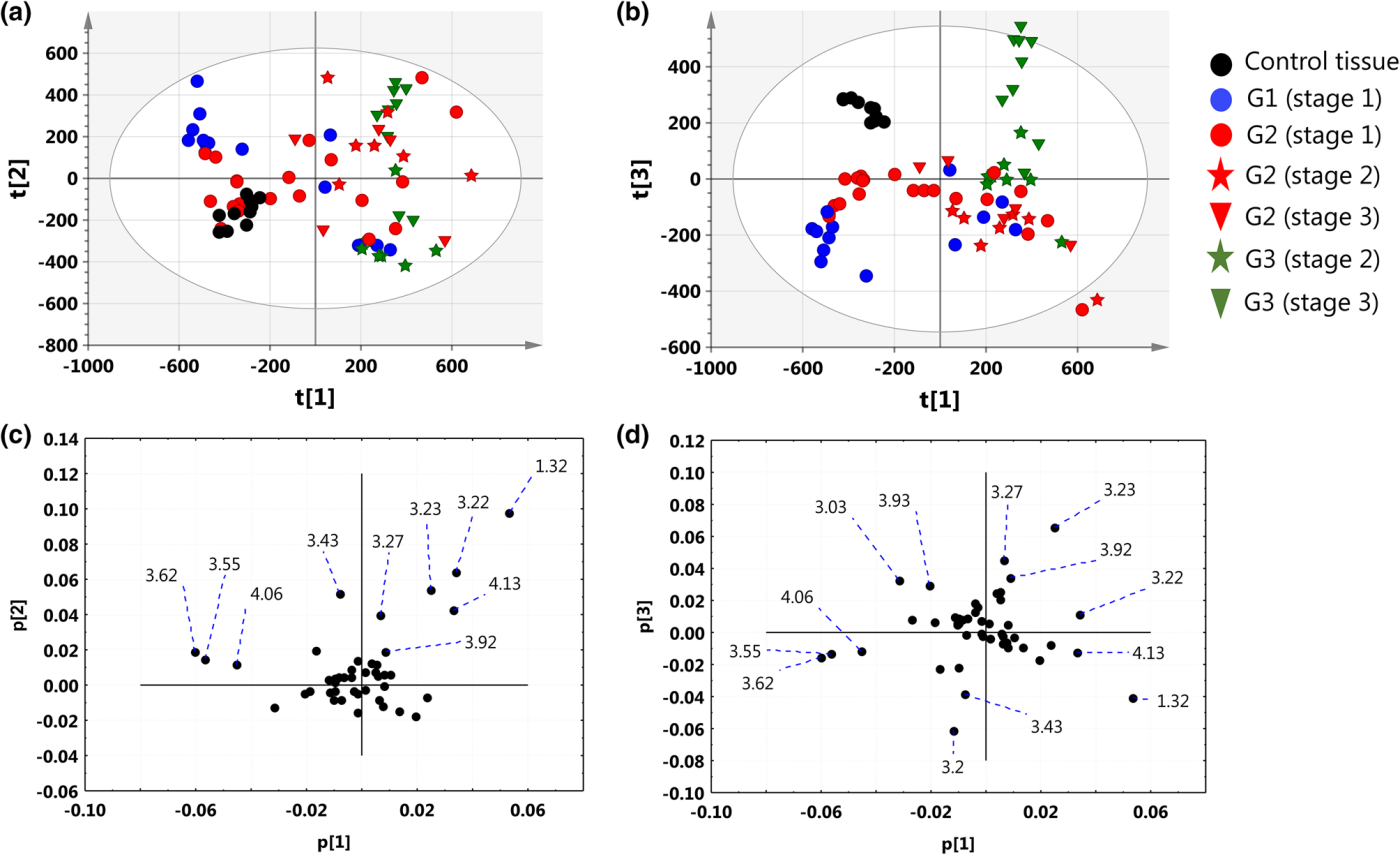
The PCA scores plots obtained from the model 1 (in the parentheses there are the percentage values of the total variation in the given projection plane) for: (**a**) PC1–PC2 (44.2%), (**b**) PC1–PC3 (35.41%). The loadings plots for: (**c**) PC1–PC2, (**d**) PC1–PC3. The points in the scores plots are marked according to the disease stage and tumors grade, while the points in the weights plots are labeled according to the chemical shifts. For clarity, the most important chemical shifts are presented. The scores plots (**a**,**b**) were created using SIMCA-P 15.0 software package (https://www.sartorius.com). The loadings plots (**c**,**d**) were created using Statistica 12.5 software (www.statsoft.pl) based on data exported from SIMCA-P 15.0 software package (https://www.sartorius.com).

The results obtained from the unsupervised PCA analysis (model 1) are presented in Fig. 3. Although a substantial overlap between the different tumor grades and the disease stages is seen in the PC1-PC2 projection plane, the majority of the G1 (stage 1) and G2 (stage 1) tumors were assigned the negative PC1 scores, while the G2 (stage 2 + 3) and G3 (stage 2 + 3) tumors were characterized by the positive PC1 scores (Fig. 3a). The direction of the largest variation in the dataset is mainly influenced by the positive contributions of lactate (1.32 ppm, 4.13 ppm), phosphocholine (3.22 ppm) and glycerophosphocholine (3.23 ppm) and the negative contribution of myo-inositol (3.55 ppm, 3.62 ppm, 4.06 ppm) (Fig. 3c). The discrimination of the G1, G2 and G3 tumors from the non-malignant tissue and a quite good separation between the G3 tumors and the remaining grades is apparent in the PC1-PC3 projection plane (Fig. 3b). The loadings plot indicates that high creatine (the signals at 3.03 and 3.93 ppm) and low lactate (the signals at 1.32 and 4.13 ppm) are the most characteristic metabolic features for the control tissue. The separation of the G3 tumors from the remaining samples is mainly caused by high GPC (3.23 ppm) and betaine (3.92 ppm, 3.27 ppm) and low choline (3.20 ppm) in this group (Fig. 3d).

### Supervised three-class discrimination according the to the tumor grade and the disease stage

**Figure 4:**
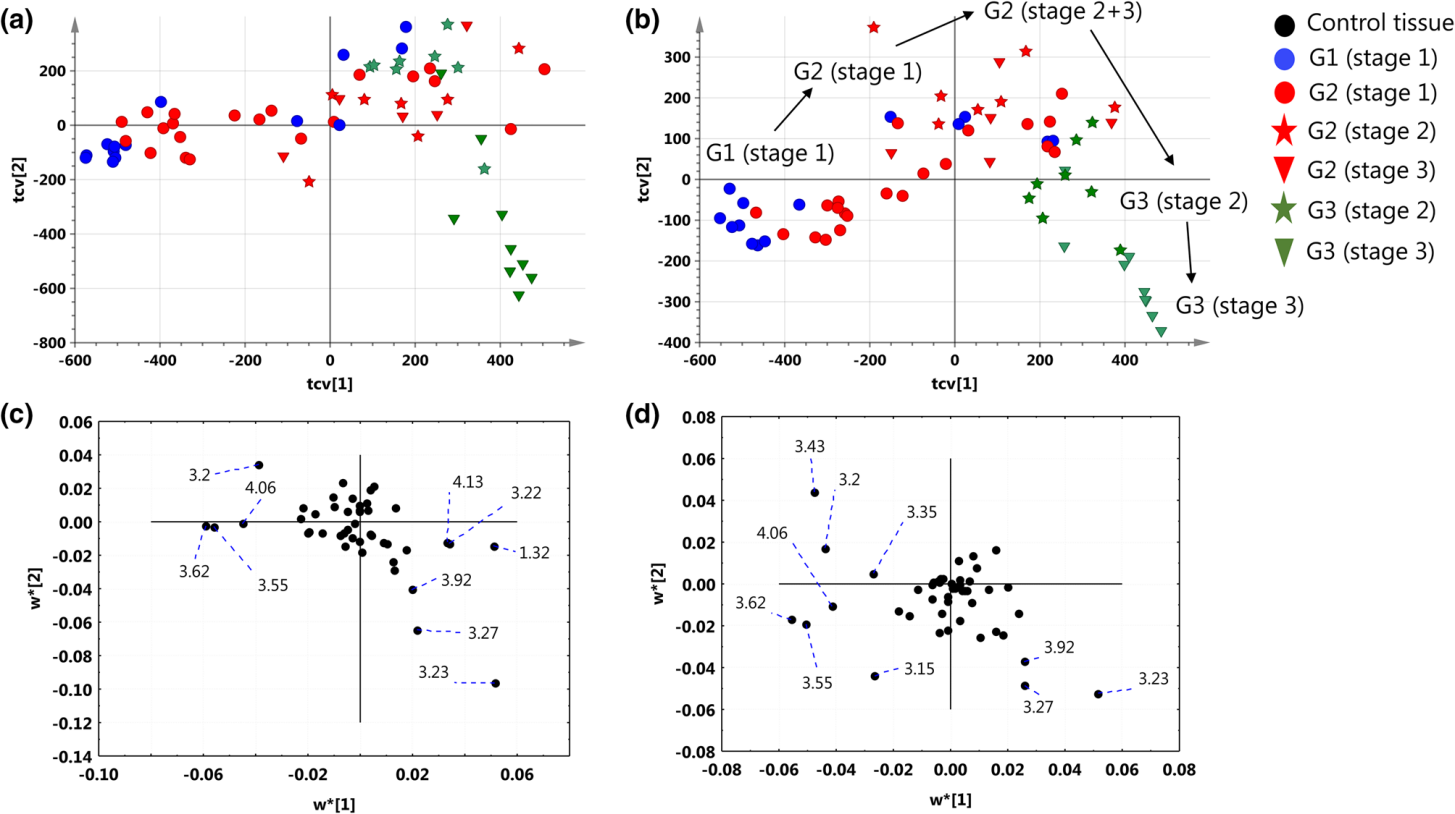
The cross-validated PLS-DA scores (**a**) and weights (**c**) plots obtained from the model 2 differentiating the patients according to the disease stage (2 components, R2X = 44.1%, R2Y = 44.6%, Q2 = 36.2%). The cross-validated PLS-DA scores (**b**) and weights (**d**) plots obtained from the model 3 differentiating the patients according to the tumor grade (2 components, R2X = 44.8%, R2Y = 70.4%, Q2 = 46.7%). The points in the scores plots are marked according to the disease stage and tumors grade, while the points in the weights plots are labeled according to the chemical shifts. For clarity, the most important chemical shifts are presented. The scores plots (**a**,**b**) were created using SIMCA-P 15.0 software package (https://www.sartorius.com). The weights plots (**c**,**d**) were created using Statistica 12.5 software (www.statsoft.pl) based on data exported from SIMCA-P 15.0 software package (https://www.sartorius.com).

The cross-validated scores and loadings plots obtained from the PLS-DA models differentiating different disease stages (model 2) and tumor grades (model 3) are presented in Fig. 4. Although the majority of the patients diagnosed with stage 1 were assigned the negative PC1 scores, a considerable overlap between the different disease advancements in the first quadrant of the scores plot can be observed (Fig. 4a). The most aggressive tumors in the dataset [G3 (stage 3)] form a distinct cluster in the fourth quadrant of the plot. The high levels of myo-inositol (4.06 ppm, 3.62 ppm, 3.55 ppm) and choline (3.20 ppm) and the low levels of lactate (4.13 ppm, 1.33 ppm) and phosphocholine (3.22 ppm) are characteristic for the G1 (stage 1) and G2 (stage 1) tumors, while the G3 (stage 3) tumors present with the increased GPCho (3.23 ppm) and betaine levels (3.27 ppm, 3.92 ppm) (Fig. 4c). There is an interesting trajectory reflecting the tumor grade visible in the scores plot obtained from the other model (Fig. 4b). Within the pathomorphological grade group a sub-clustering of the patients diagnosed with different stages is visible. Apart from high myo-inositol in the G1 (stage 1) and G2 (stage 1) tumors and high GPCho and betaine in the G3 (stage 3) tumors the weights plots for the model 3 reveals additionally the lowest taurine (3.43 ppm) level in the G3 tumors and the high signal of dimethyl sulfone (3.15 ppm) in the G1 tumors (Fig. 4d).

### Pair-wise discrimination between the endometrial cancer of different grades and healthy endometrium

**Figure 5:**
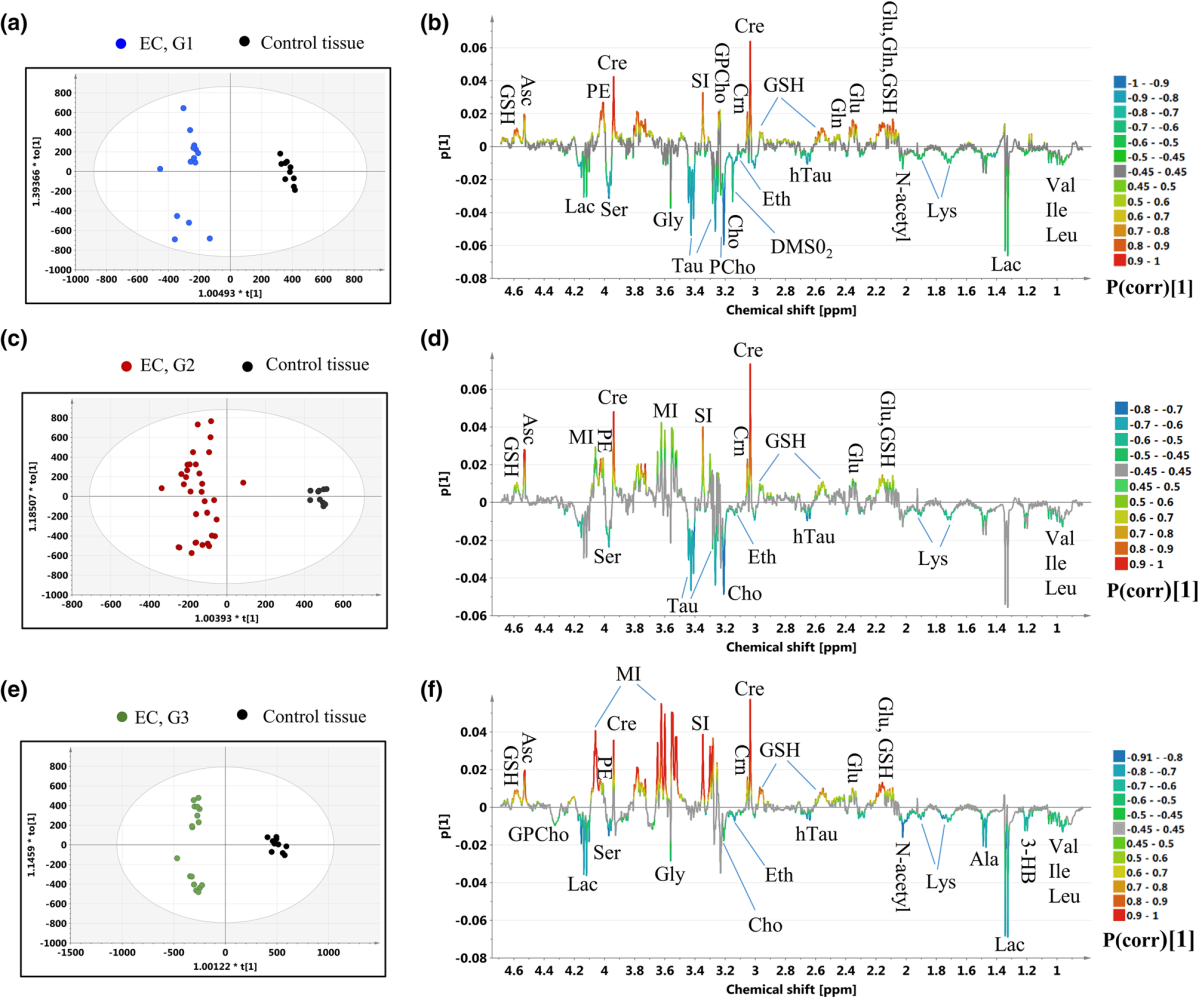
The OPLS-DA models: (**a**) the scores plot and (**b**) the loadings plot for the model 4, (**c**) the scores plot and (**d**) the loadings plot for the model 5, (**e**) the scores plot and (**f**) the loadings plot for the model 6. The color circles represent: black dot—a control tissue, blue dot—G1 EC, red dot—G2 EC, green dot—G3 EC. *Val* valine, *Ile* isoleucine, *Leu* leucine, *3-HB* 3-hydroxybutyrate, *Lac* lactate, *Ala* alanine, *Glu* glutamate, *Gln* glutamine, *GSH* glutathione, *Lys* lysine, *N-acetyl* N-acetyl compound, *hTau* hypotaurine, *Cre* creatine, *Crn* creatinine, *PCho* phosphocholine, *GPCho* glycerophosphocholine, *Cho* choline, *Eth* ethanolamine, *SI* scyllo-inositol, *Tau* taurine, *Gly* glycine, *MI* myo-inositol, *PE* phoshoethanolamine, *Ser* serine, *Asc* ascorbate. The image was created using SIMCA-P 15.0 software package (https://www.sartorius.com).

Figure 5 presents the results obtained from the OPLS-DA analyses constructed to differentiate the G1, G2 and G3 tumors from the healthy endometrium (the models 4, 5 and 6, respectively). The number of the model components, the fractions of the X and Y variation explained by the predictive and orthogonal components (R2X and R2Y), the fractions of the Y variation predicted by the models (Q2), the p values from the CV-Anova test and the results from the permutation testing are presented in Table S2. The p(corr)[1] and VIP values obtained from the multivariate analysis, the results from the univariate statistical assessment (the fold-changes and the p values) for the metabolites contributing to the class separation are listed in Table S3.

The OPLS-DA analysis reveals the increased valine, isoleucine, leucine, hypotaurine, serine, lysine, ethanolamine, choline and decreased creatine, creatinine, glutathione, ascorbate, glutamate, phosphoethanolamine and scyllo-inositol in the endometrial tumors of all grades in reference to the non-transformed tissue. These results are in a general agreement with the findings obtained from the univariate analysis. However, the Kruskal–Wallis test followed by multiple comparisons of the mean ranks indicates the lack of a statistical significance of the differences in the choline levels between the G3 tumors and the normal endometrium, and the isoleucine levels between the G1 tumors and the non-transformed tissue (Table S3). Although the univariate analysis shows that the N-acetyl compound is increased in the tumors of all grades relative to the healthy endometrium, the |p(corr)[1]| values for the G2 tumors are slightly lower than 0.4. The multivariate analysis shows increased phosphocholine and decreased glycerophosphocholine (3.23 ppm) in the G1 tumors. These results are in agreement with the univariate analysis of the integrals derived from the line fitting. Although the phosphocholine and glycerophosphocholine signals were assigned the |p(corr)[1]| values not exceeding 0.45 in the model differentiating the G2 tumors from the normal endometrium, the univariate analysis of the integrals obtained from line fitting provides the similar results as for the G1 tumors. The multivariate analysis indicates the increase of the signal at 4.33 ppm (corresponding to glycerophosphocholine) in the G3 tumors relative to the normal tissue, which is, however, not confirmed by the univariate statistics. Both approaches revealed increased taurine in the G1 and G2 tumors and elevated alanine in the G3 tumors as compared to the healthy tissue. Alanine was also found to be significantly increased in the G2 tumors in the univariate analysis. In turn, OPLS-DA shows the glycine level to be higher in the G1 and G3 tumors in comparison to the non-cancerous tissue, while the univariate analysis revealed the trends towards the increased glycine levels in these tumors (0.06 < p < 0.08). The OPLS-DA technique shows myo-inositol in the G2 and G3 tumors to be decreased relative to the value for the non-transformed endometrium, while the univariate analysis indicates that this change is specific for the G3 tumors. According to the results obtained from the OPLS-DA models, in the G1 and G3 tumors the lactate levels are increased, while the p(corr)[1] values for the G2 tumors are slightly lower than 0.45. The higher lactate levels (at 1.33 ppm) in the G2 and G3 tumors are also confirmed by the univariate analysis. The multivariate analysis reveals increased 3-hydroxybutyrate in the G3 tumors in comparison to the healthy tissue, while in the univariate approach this change is also observed for the G2 tumors. Although the univariate analysis shows a downward glutamine trend in endometrial tumors irrespective of tumor grade (p ~ 0.07), OPLS-DA shows this metabolite to be reduced only in the G1 tumors. Of note, the singlet at 3.15 ppm assigned to dimethyl sulfone was observed in the majority of the spectra acquired from the G1 tumors. This signal was not observed in the specimens obtained from the G2 and G3 tumors and from the healthy tissue. On the other hand, the signals at 3.27 and 3.92 ppm (corresponding to betaine) were detected in the spectra measured from the G3 tumors. Although the univariate analysis did not show the betaine levels to statistically differ between these tumors and the healthy tissue, the |p(corr)[1]| parameter reached the value of 0.45 for the signal at 3.92 ppm. The Venn diagram showing the number of differentiating metabolites (common and unique) to each tumor grade in reference to the control tissue is presented in the Supplementary materials (Figure S2).

### Analysis of the shared and distinct metabolic features of the G2 tumors (stage 1) and the G2 tumors (stages 2 + 3) in reference to the control tissue

The results obtained from the OPLS-DA models S1 [G2(stage 1) vs control tissue] and S2 [G2 (stage 2 + 3) vs control tissue] are presented in the Supplementary Material (Figures S4, S5; Table S8). The SUS plot analysis (Figure S6) indicates that the majority of the metabolic changes in the G2 tumors in reference to the healthy endometrium revealed by the OPLS-DA model 5 are shared between the G2 (stage 1) and G2 (stage 2 + 3) tumors. Specifically, the common features include: increased isoleucine, leucine, valine, lysine, taurine, serine, hypotaurine, choline and ethanolamine, and decreased glutamate, creatinine, glutathione, scyllo-inositol, creatine, phosphoethanolamine and ascorbate in the tumors than in the healthy tissue. The separate OPLS-DA models S1 and S2 revealed also an increase in 3-hydroxyburyate and N-acetyl compound in the G2 tumors, irrespective of the disease stage. Increased lactate, alanine, and phosphocholine and decreased glucose, myo-inostol and glutamine and acetate were found to be characteristic for the G2 (grade 2 + 3) group in relation to the non-transformed tissue [not observed in the G2 (grade 1) group], while lower succinate was distinctly observed in the G2 (grade 1) group (Table S9).

### Discrimination between different grades of endometrial cancer

OPLS-DA was exploited to examine the metabolic differences between the endometrial cancers of different grades (models 7–9). The models diagnostics (R2X, R2Y, Q2, the p values from the CV-Anova test and the results from the permutation testing) are presented in Table S4.

#### Model 7 (Grade 1 endometrial cancer vs. grade 2 endometrial cancer)

**Figure 6:**
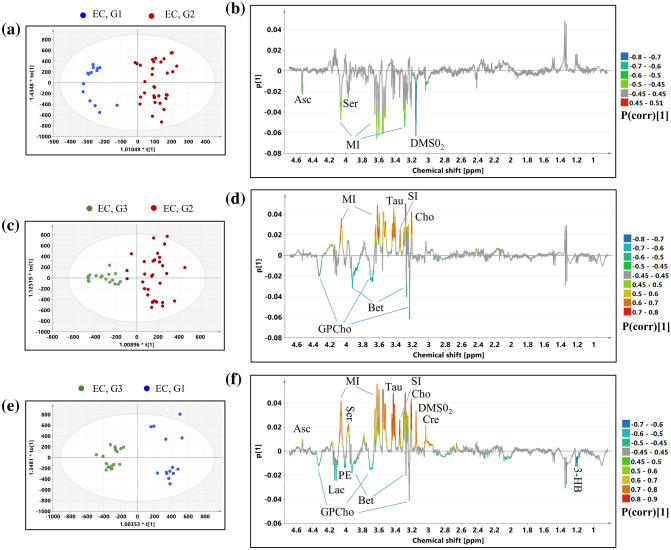
Results obtained from the OPLS-DA models: (**a**) the scores plot and (**b**) the loadings plot for the model 7, (**c**) the scores plot and (**d**) the loadings plot for the model 8, (**e**) the scores plot and (**f**) the loadings plot for the model 9. The color circles represent: blue dot—G1 EC, red dot—G2 EC, green dot—G3 EC. *3-HB* 3-hydroxybutyrate, *Cre* creatine, *GPCho* glycerophosphocholine, *Cho* choline, *DMSO*_*2*_ dimethyl sulfone, *SI* scyllo-inositol, *Tau* taurine, *MI* myo-inositol, *PE* phoshoethanolamine, *Ser* serine, *Bet* betaine, *Asc* ascorbate. The image was created using SIMCA-P 15.0 software package (https://www.sartorius.com).

The scores and loadings plots obtained from the OPLS-DA model 7 differentiating the G1 tumors from the G2 tumors are presented in Fig. 6a,b. The p(corr)[1] values, the VIP scores and the results from the univariate statistical assessment (the fold-changes and the p values) for the metabolites contributing to the class separation and the AUC values for the individual metabolites are listed in Table S5. The higher levels of serine, dimethyl sulfone, myo-inositol, ascorbate in the G1 tumors than in the G2 ones are mainly responsible for the observed separation. The AUC values for these metabolites were found to be above 0.7. However, in case of myo-inositol the difference was not statistically significant in the univariate analysis (Table S5). The analysis of the ROC curve for the predicted Y scores reveals AUC of 0.959, sensitivity of 0.909 and specificity of 0.929 (Figure S1a, the Supplementary materials).

The changes of serine, dimethyl sulfone and ascorbate were also observed in the G2 (stage 1) tumors when compared to the G1 tumors (O-PLS-DA model S3, Figure S7, Table S10, Supplementary materials). Additionally, lower succinate and taurine were observed in G2 (stage 1) than in G1 (stage 1). These changes were not visible before stage matching and are characterized by the AUC value ca. 0.7.

#### Model 8 (Grade 2 endometrial cancer vs. grade 3 endometrial cancer)

The OPLS-DA analysis conducted to find the metabolic differences between the G2 and G3 tumors resulted in the model 8 composed of 1 predictive and 2 orthogonal components (Table S4). Table S6 presents the p(corr)[1]|values, the VIP scores and the p values obtained from the univariate analysis. The scores and loadings plots are presented in Fig. 6c,d. The higher glycerophosphocholine and betaine along with the lower choline, scyllo-inositol, taurine and myo-inoitol in the G3 tumors in comparison to the G2 tumors are responsible for the clustering visible in the scores plot (Fig. 6c). These metabolites were also shown to be statistically different between the groups in the univariate analysis and are characterized by AUC values > 0.7. (Table S6). The analysis of the ROC curve for the predicted Y scores reveals AUC of 0.998, sensitivity of 1.0 and specificity of 1.0 (Figure S1b, Supplementary materials).

The OPLS-DA model S4 differentiating the G2 grade (stage 2 + 3) and G3 grade (stage 2 + 3) tumors shows similar metabolic changes between the tumor grades (Figure S8, Table S11). The AUC values for these metabolites were found to be similar before (Table S6) and after stage matching (Table S11). However, the analysis of the stage matched G2 and G3 tumors reveals additionally increased succinate and creatine, and lower ascorbate and glucose in the G2 tumors than in the G3 grade ones. Of them, only succinate and glucose are characterized by the AUC values above 0.7.

#### Model 9 (Grade 1 endometrial cancer vs. grade 3 endometrial cancer)

The results obtained from the OPLS-DA model 9 constructed to differentiate the G1 tumors from the G3 tumors are shown in Fig. 6e,f. The p(corr)[1] values, the VIP scores and the univariate analysis results are presented in Table S7.

The separation visible in the scores plot is mainly caused by higher 3-hydroxybutyrate, phosphoethanolamine, lactate, betaine, and lower ascorbate, serine, myo-inositol, taurine, scyllo-inositol, choline, dimethyl sulfone and creatine in the G3 tumors in comparison to the G1 ones. These changes were also statistically significant in the univariate analysis, with the exception of phosphoethanolamine, lactate, creatine and ascorbate (Table S7). Although the |p(corr)[1]| value for the glycerophosphocholine signal at 3.23 ppm is slightly below 0.45, the univariate analysis of the line fitted integrals shows that the increase of this metabolite in the G3 tumors in comparison to the G1 ones is at the border of a statistical significance (p = 0.059). The changes in the remaining signals (3.70 ppm, 4.33 ppm) corresponding to this metabolite confirm this trend. The analysis of the ROC curve for the predicted Y scores reveals AUC of 1.0, sensitivity of 1.0 and specificity of 1.0 (Figure S1c, Supplementary materials).

It was not possible to analyze the stage matched G1 and G3 tumors in our work.

### SUS plot analysis—comparison between the OPLS-DA models 7 and 8

**Figure 7:**
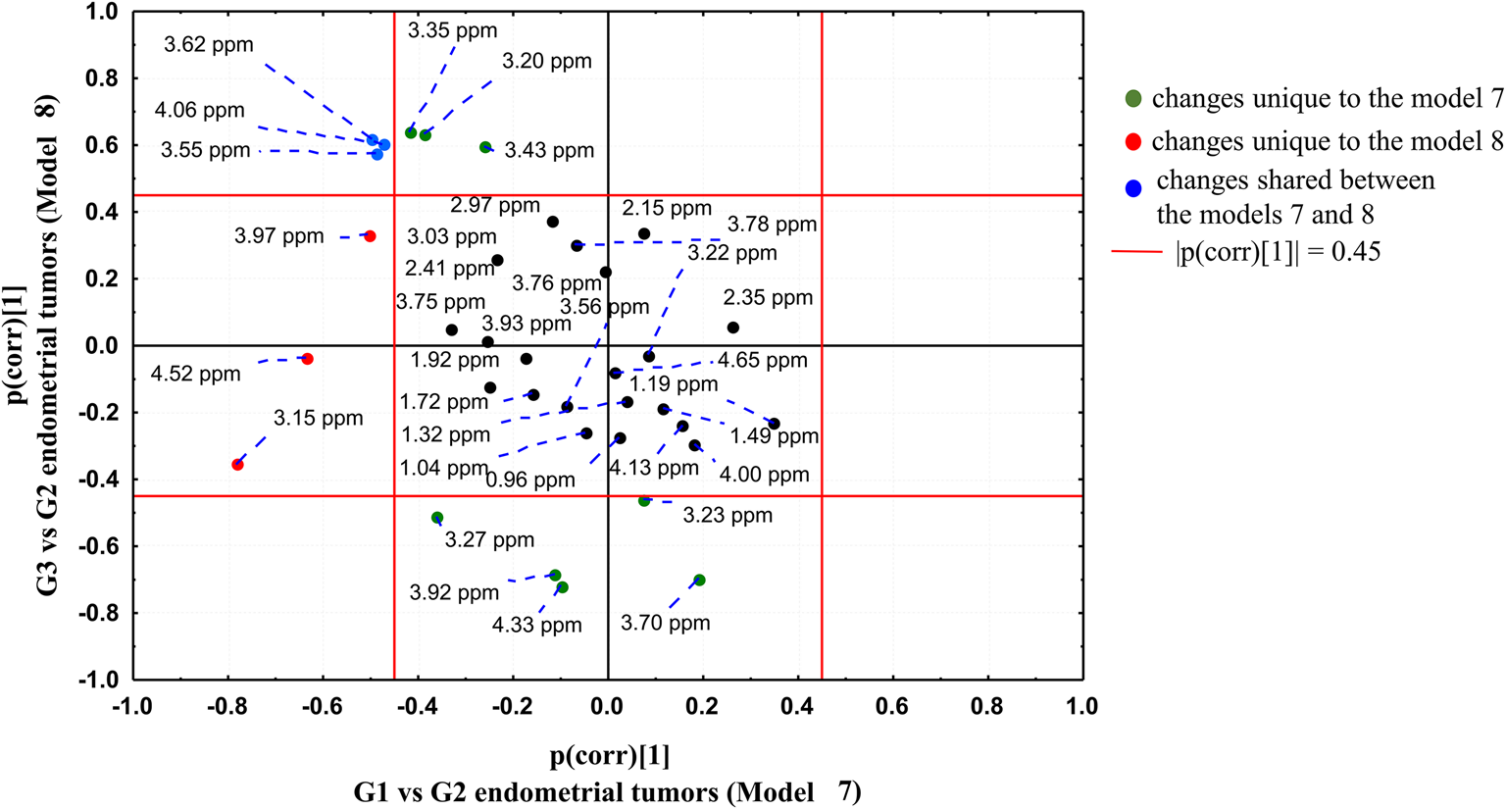
SUS plot comparing the OPLS-DA models 7 (discriminating the G1 and G2 tumors) and 9 (discriminating the G3 and G2 tumors). The image was created using Statistica 12.5 software (www.statsoft.pl).

The OPLS-DA models 7 and 8 were compared to each other by means of the SUS plot (Fig. 7) in order to find the specific and common metabolic features of the G1 and G3 tumors in comparison to the tumors of the intermediate G2 grade.

Higher dimethyl sulfone (3.15 ppm), ascorbate (4.52 ppm) and serine (3.97 ppm) are the metabolic features specific to the G1 tumors in comparison to the G2 ones (not observed in G3). These metabolites are located in the SUS plot area characterized by |p(corr)[1]|> 0.45 in the model 7 and |p(corr)[1]|< 0.45 in the model 8. The univariate analysis confirms the specificity of the change for these metabolites (p values < 0.05 obtained for the comparison of the G1 to G2 tumors and the lack of a statistical significance for the comparison of the G3 to G2 tumors, Tables S5, S6). In the G3 tumors glycerophosphocholine (3.23 ppm, 3.70 ppm, 4.33 ppm) and betaine (3.27 ppm, 3.92 ppm) are higher and taurine (3.43 ppm), scyllo-inositol (3.35 ppm) and choline (3.20 ppm) are lower than in the G2 ones (not observed in G1 tumors). These results are the same in both multivariate and univariate analyses. The chemical shifts corresponding to myo-inositol (4.06 ppm, 3.62 ppm, 3.55 ppm) are located near the negative diagonal in the SUS plot, suggesting the opposite behavior of this metabolite in the G1 and G3 tumors in reference to the intermediate G2 grade. Although the univariate analysis reveals a statistical significance of the difference between the G2 and G3 tumors and the lack of a significance for a comparison of G1 to G2, a downward change of the median myo-inositol level according to the degree of differentiation can be observed (Tables S5, S6).

### Analysis of metabolic pathways enrichment and impact

**Figure 8:**
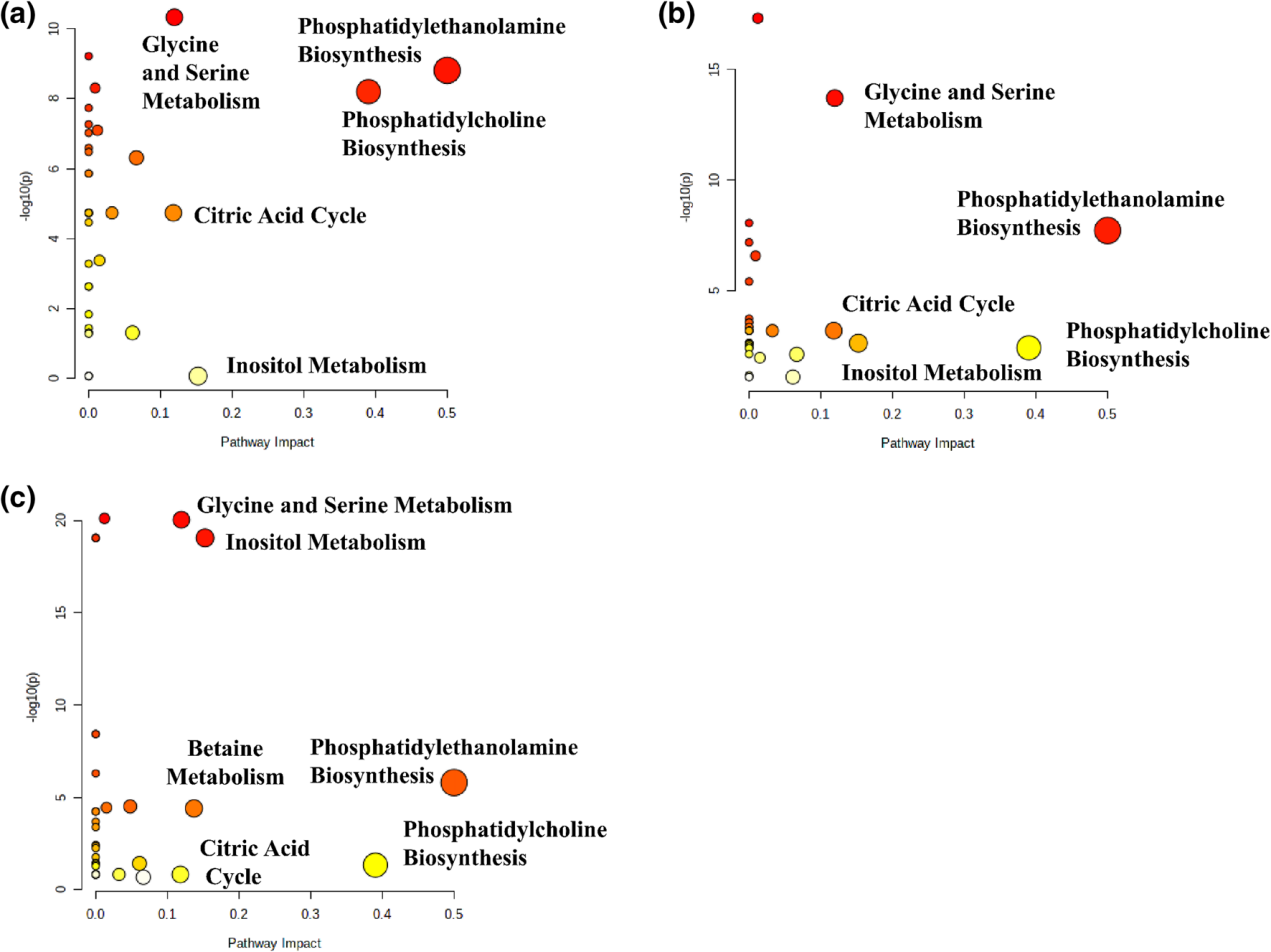
Metabolome views obtained from the pathways analysis presenting the pathways altered in (**a**) G1 vs. healthy tissue, (**b**) G2 vs. healthy tissue, (**c**) G3 vs. healthy tissue. The image was created using Metaboanalyst 4.0 software (https://www.metaboanalyst.ca).

The metabolome views obtained from the analysis of the metabolic differences between the different EC grades and the normal tissue are presented in Fig. 8, while the fold enrichments obtained from the enrichment analysis for the most important metabolic pathways are shown in Table 2. Phosphatidylethanolamine biosynthesis as well as glycine and serine metabolism are disturbed in endometrial cancer of all grades in comparison to the normal endometrium (impact scores > 0.1, Holm p values < 0.05). The statistically significant alteration of phosphatidylcholine biosynthesis is observed for the G1 tumors. Although this pathway is also characterized by the relatively large impact scores for the G2 and G3 tumors, the borderline Holm p value (0.09) was reached only for the G2 tumors. The fold enrichment scores for the inositol metabolism were found to increase with grade. The requirement of the Holm p value < 0.05 was fulfilled for the G3 tumors, while the G2 tumors were characterized by the borderline p value (0.08) for this pathway. On the contrary, a decrease of the citric acid cycle fold enrichment scores as a function of grade is also apparent. The betaine metabolism pathway was found to be perturbed exclusively in the G3 tumors.

**Table 2:** Fold enrichment values (and the Holm p values) obtained from the enrichment analysis for the most important metabolic pathways (impact > 0.1) perturbed in the G1, G2 and G3 endometrial tumors in reference to the non-tumoral tissue.

Metabolic pathway	G1 vs. normal endometrium	G2 vs. normal endometrium	G3 vs. normal endometrium
Phosphatidylcholine biosynthesis	**60.83 (3.34E−07)**	16.64 (8.73E**−**02)	12.16 (7.46E**−**01)
Phosphatidylethanolamine biosynthesis	**77.07 (8.82E−08)**	**43.53 (1.09E−06)**	**45.53 (7.44E−05)**
Citric acid cycle	**57.32 (5.90E−04)**	**24.89 (2.71E−02)**	8.22 (1.0)
Inositol metabolism	0.15 (1.0)	20.32 (7.73E**−**02)	**96.54 (5.17E−18)**
Glycine and serine metabolism	**55.32 (1.65E−10)**	**38.87 (2.49E−15)**	**60.50 (4.56E−20)**
Betaine metabolism	–	–	**29.92 (1.52E−03)**

The bolded fold enrichment scores indicate the metabolic pathways characterized by the Holm p value < 0.05.

**Figure 9:**
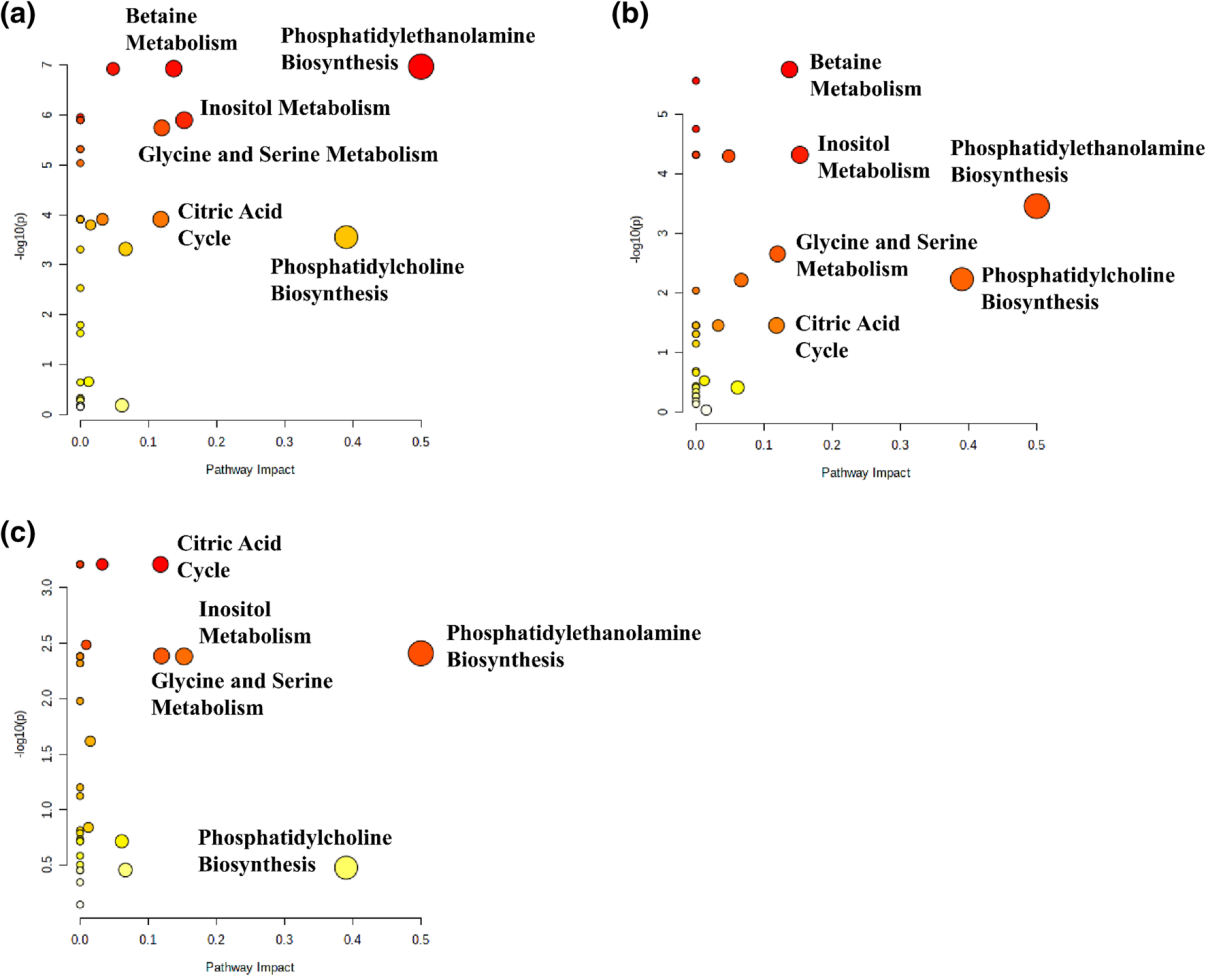
Metabolome views obtained from the pathways analysis presenting the pathways altered in EC: (**a**) G1 vs. G3, (**b**) G2 vs. G3 (**c**) G1 vs. G2. The image was created using Metaboanalyst 4.0 software (https://www.metaboanalyst.ca).

The metabolome views obtained from the analysis of the metabolic differences between the endometrial tumors of different grades are presented in Fig. 9, while the fold enrichments obtained from the enrichment analysis for the most important metabolic pathways are shown in Table 3. The largest perturbations are observed for the G1 to G3 tumors comparison (Fig. 8a). The significant alterations (impact score > 0.1, Holm p value < 0.05) of phosphatidylethanolamine biosynthesis, inositol and betaine metabolism are seen also for the comparison of the G2 to G3 tumors (Fig. 8b). For the comparison of the G1 to G2 tumors (Fig. 8c) the citric acid cycle is the only significantly changed metabolic pathway.

**Table 3:** Fold enrichment values (and the Holm p values) obtained from the enrichment analysis for the most important metabolic pathways perturbed in EC: G1 vs. G2, G1 vs. G3 and G2 vs. G3.

Metabolic pathway	G1 vs. G2 tumors	G1 vs. G3 tumors	G2 vs. G3 tumors
Phosphatidylcholine biosynthesis	2.10 (1.0)	**29.80 (1.15E−02)**	13.66 (2.93E**−**01)
Phosphatidylethanolamine biosynthesis	13.67 (2.02E**−**01)	**51.67 (6.47E−06)**	**16.45 (1.77E−02)**
Citric acid cycle	**23.16 (3.69E−02)**	**40.37 (5.78E−03)**	8.92 (1.0)
Inositol metabolism	16.85 (2.12E**−**01)	**56.03 (7.05E−05)**	**29.39 (2.68E−03)**
Glycine and serine metabolism	8.26 (6.48E**−**01)	**22.08 (3.48E−03)**	6.37 (8.07E**−**01)
Betaine metabolism	–	**55.10 (6.99E−06)**	**32.50 (1.06E−04)**

The bolded fold enrichment scores indicate the metabolic pathways characterized by the Holm p value < 0.05.

Interestingly, similar metabolic pathways were found to be disturbed before (Fig. 9) and after (Figure S9, supplementary materials) stage matching for the comparison of G1 vs. G2 and G2 vs G3 tumors.

## Discussion

The metabolic profiles of the endometrial cancers were found to be dysregulated as compared to the healthy endometrium and the metabolic diversity between the EC histological grades and stages was characterized. The NMR biomarkers that illustrate the severely altered endometrial cancer phenotypes can be classified as the proliferation-associated markers (e.g., various amino acids, which are indicative of proliferative activity), maintenance of intracellular redox homeostasis (glutathione), osmoregulation (inositols, taurine), and glucose and lipid metabolism (lactate, free Cho, PCho, GPCho, lipids).

The role of the identified metabolites was elucidated basing upon the metabolic pathways enrichment (the Holm p values) and the pathway topology analysis (pathway impact). The metabolic pathways, specified by a series of enzyme-catalyzed biochemical reactions that bring a number of metabolites together, can be combined with the disease processes—as a cause and effect relationship—reflected by some small-molecule metabolites' dysregulations being the primary hallmark of disease or a sign of its progression. On such ground the pathway analysis was used to visualize the differences in tumor metabolism between the EC grades and the healthy tissue and between the individual grades (Figs. 8, 9, respectively). As revealed, the statistically important metabolites can be grouped in 4–5 pathways of the different impact and role, depending on the grade.

Phosphatidylethanolamine and phosphatidylcholine biosynthesis were identified as the important pathways dysregulated in endometrial cancer relative to the normal tissue and between the tumors of different grades.

Phosphatidylcholine (PtdCho) is the most abundant phospholipid in mammalian cell membranes. There are two pathways of its de novo synthesis: the CDP-choline pathway, also known as the Kennedy pathway, and an endogenous pathway via three sequential methylations of phosphatidylethanolamine by phosphatidylethanolamine *N*-methyltransferase (PEMT). The former one is the major route, the latter is significant only in the liver^30^. The choline containing metabolites: free choline (Cho), glycerophosphocholine (GPCho), and phosphocholine (PCho) are the important water-soluble intermediates in phosphatidylcholine metabolism. Activation of choline metabolism is a critical step of cancer development and leads to increased levels of phosphocholine, GPCho and the total choline-containing compounds in various cancers^31,32^. Until recently, most studies have focused on the elevated PCho levels in cancer, while GPCho has remained a poorly understood oncometabolite. However, Sonkar and colleagues^33^ have lately published a comprehensive NMR-based review about the GPCho pathway and its role in cancer biology, but such observations are sparse in case of EC. High-resolution MAS NMR spectroscopy applied in the study comparing the endometrial tissues from 10 G3 endometrioid EC cases and 10 benign controls showed the increased PCho as an EC marker^25^. In some cancer cell types, such as isolated breast and ovarian cancer cells, a relative decrease of GPCho was observed as well, which led to the suggestion to use a higher ratio of PCho/GPCho as a marker of tumor progression^31^. The total cholines (Cho, PCho and GPCho) level detected by ^1^H MRS in vivo was shown to be associated with the endometrial tumor grade^34^, whereas the elevations of PCho in the blood^35^ and in the cervicovaginal fluid^36^ were reported at an early stage of tumor development. We found the elevated total choline containing compounds in the tumoral tissue as compared to the healthy endometrium, albeit with no association to tumor malignancy (not shown). In our study the largest alterations of phosphatidylcholine metabolism were detected for the G1 tumors (Fig. 8, Table 2). The perturbations of this pathway were also at the border of a statistical significance for the G2 tumors (the Holm p value of 0.09), whereas in the G3 tumors its role is negligible (Table 2). This is in contrast to the result by Trousil et al.^25^ who reported a strong increase in the phosphocholine levels in EC. Although in their small sample study the GPCho levels in the G3 tumors and in the healthy endometrium were found to be statistically similar, the expression of catabolic lysophospholipase enzyme forming this metabolite was increased. The supervised PLS-DA analysis performed in our study revealed that the high GPCho level was characteristic for the G3 (stage 3) tumors (Fig. 4). This metabolic feature is also important for the differentiation of the G3 tumors from the remaining grades (OPLS-DA models 8 and 9). Moreover, the univariate and/or multivariate statistics revealed GPCho to be lower in the G1 and G2 tumors in reference to the normal tissue (Table S3). Similar tendencies were also reported for breast and ovarian cancers^37,38^.

The OPLS-DA analysis shows also elevated choline in the cancerous samples in comparison to the healthy endometrium, irrespectively on the tumor grade (Fig. 5, Table S3). In the high grade G3 tumors this metabolite was found to be less abundant than in the tumors of the lower grades.

Although Trousil et al.^25^ did not report alterations in the choline level in the G3 tumors, the age difference between the endometrial cancer patients and the control group (implicating a difference in the menopausal status) could be a significant confounder in their work. The recent serum metabolomics study identified sphingomyelins and phosphatidylcholines as key metabolites differentiating pre- from post-menopausal women^39^. The menopausal status was found to be associated with higher dietary requirement for choline^40^. Interestingly, the choline levels in endometrial tumors measured with the use of in vivo ^1^H MRS are higher in younger patients (age below 66 years) than in older ones^34^. Our preliminary results also indicate that in the post-menopausal women the choline levels are markedly lower while the phosphoethanolamine (involved in sphingomyelin metabolism) levels are higher than in the pre-menopausal ones (the data not shown).

Phosphoethanolamine is another phospholipid (a precursor in the synthesis of phosphatidylethanolamine) being deregulated in cancer. Most often, its levels are increased^41,42^, however, the reduced phosphoethanolamine synthesis was reported in mutant isocitrate dehydrogenase 1 (IDHmut) gliomas relative to wild-type IDH1 gliomas^43^. In humans, de novo formation of phosphatidylethanolamine occurs via several pathways, but the Kennedy pathway plays a central role in the synthesis. Our findings show that in the endometrial cancers of all grades the levels of ethanolamine are increased and those of PE are decreased in comparison to the non-transformed tissue (Fig. 5, Table S3). Moreover, the multivariate analysis revealed the higher phosphoethanolamine level in the G3 tumors than in the G1 ones (Fig. 6, Table S7). This metabolite is also synthesized from phosphatidylserine by phosphatidylserine decarboxylase—and its biosynthesis is regulated by serine. In fact, we observe the alterations in the levels of serine in the EC samples of all grades.

Serine participates also in serine and glycine metabolism. As reveals from the OPLS-DA analysis (Fig. 5) endometrial cancer is characterized by the higher serine (the G1, G2 and G3 tumors) and glycine (the G1 and G3 tumors) levels than the non-transformed tissue. The serine fold changes obtained from the univariate statistics and the p(corr)[1] values derived from OPLS-DA decrease with the tumor grade (Table S3). Serine and glycine metabolism was found to be particularly important in discrimination between the G1 and G3 tumors. This pathway involves the synthesis and breakdown of several small amino acids (including glycine, serine, and cysteine). Serine and glycine, not essential amino acids, can be synthesized from several routes, and provide the essential precursors for the synthesis of proteins, nucleic acids and lipids—the crucial materials also for cancer cell growth^44^. They are also required for the maintenance of cellular redox state. Their crucial contribution is through the glycine cleavage system, which refuels one-carbon metabolism—a complex cyclic metabolic network based on the chemical reactions of folate compounds (glycine is an integral component of glutathione, the main antioxidant molecule of the cell)^44^ and through the serine catabolism, maintaining the mitochondrial redox balance during hypoxia^45^. Therefore, this metabolic pathway has a pivotal role in proliferating cells, including cancer cells, and its hyper-activation is claimed to drive tumorigenesis^46,47^. Our results seem to indicate that the serine and glycine changes observed for the EC grades and seen in the OPLS-DA models result from the interplay of the consumption versus production of these amino acids in the cancer cells of increasing proliferative activity. As shown by Labuschagne et al.^48^ studying the cancer cells models, there is a correlation between glycine uptake and proliferation rate, reflecting a rapid depletion of exogenous serine. The authors concluded that glycine uptake is a consequence of rapid proliferation rather, than its cause^48,49^.

Our findings from multivariate analysis indicate that the inositol metabolic pathways are most consistently altered as compared to the healthy endometrium only in the higher EC grades, G2 and G3 (Fig. 5, Table S3). Inositol (besides the most abundant myo-inositol, the other naturally occurring and NMR-detected stereoisomer is scyllo-inositol) is produced in the human body through the conversion of glucose-6-phosphate into myo-inositol. Inside the cells, myo-inositol is present both in free form and as a component of the membrane phosphoinositides. Myo-inositol is essential or important for the smooth running of a wide range of cell functions, including cell growth and survival, has defined roles in osmoregulation, anticancer activity, and the enhancement of the anticancer effects of inositol hexaphosphate (IP6) on various cancers^50,51^. Its concentration in mammalian female reproductive tracts is substantially higher than in blood serum^52^. The polycystic ovary syndrome (PCOS) patients experience a severe deregulation of inositol metabolism, enabling the establishment of a clear mechanistic link between insulin resistance and inositol deficiency^51^. The inositol metabolic pathways were also found to be altered in highly metastatic osteosarcoma^53^ and several other reports have shown the inositol pathway metabolites to decrease with tumor progression^54,55^. Consistent with these findings, our metabolomics profiling of the EC samples revealed that the inositol pathway metabolites are significantly lower in the higher grades tumors. Myo-inositol is an end-product of lipid metabolism and gets converted to acetyl-CoA for the energy supply. Thus, a down-regulation of myo-inositol in G3 is suggestive of alterations in the lipid metabolism. The reduced levels of myo-inositol and scyllo-inositol in the G3 tumors were also observed by Trousil et al.^25^. It could also be speculated that the levels of NMR visible myo-inositol are related to the genetic alterations in the insulin-phosphoinositide 3 kinase signaling pathways observed in more than 90% of endometrial cancers^2^. Of note, this pathway has an important role in mediating the increased glucose uptake in cancer cells (Warburg effect). The metabolic shift from oxidative phosphorylation to the glycolysis could be responsible for the increased lactate level in endometrial tumors.

As reveals from our study the betaine metabolism was found to be deregulated exclusively in the G3 tumors (Fig. 5, Table S3). Betaine is not only a metabolite of choline—metabolism of both occurs in mitochondria by oxidative demethylation pathway that requires oxygen and reducing equivalents from the TCA cycle/electron transport chain^56^, but also a modified amino acid consisting of glycine with three methyl groups that serves as a methyl donor in several metabolic pathways, e.g. for the production of S-adenosyl methionine (SAM), which is a substrate for DNA and histone methyltransferases, and thus required for the establishment and maintenance of the epigenome^57,58^. Such epigenetic mechanism is well-known and plays a significant role in cancer development.

Endometrial cancer is characterized by aberrant amino acid metabolism^59^. It is also confirmed by our study—the NMR signals due to leucine, isoleucine and valine, the three branched-chain amino acids (BCAAs) are increased in endometrial tumors, especially in the G2 and G3 ones (Fig. 5, Table S3). BCAAs are used by tumors in the protein synthesis or oxidized for energy purposes. BCAAs derive from the tumor microenvironment and the protein degradation processes. The expression of the BCAA metabolic enzyme, such as the cytosolic branched-chain aminotransferase 1 (BCAT1) was reported to correlate in EC with more aggressive cancer growth and progression—it is related to tumor grade, FIGO stage and lymph node metastasis^60^. Upregulation of LAT1 transporter, enabling the passage of neutral essential amino acids (such as leucine) into the cells and the efflux of other amino acids (such glutamine), was found in endometrial cancer^61^. Recent studies indicate also upregulation of glutamine transporters (including ASCT2 and SNAT1) in this type of cancer^62^. Importantly, inhibition of glutamine transport resulted in a reduced cell growth^62^. Furthermore, the increased glutaminase expression, catalyzing the conversion of glutamine to glutamate, was reported^63^. The decreased glutamate levels observed by us in the endometrial tumors of all grades (Table S3) may be due to the increased utilization of this metabolite in endometrial cancer. Glutamate could be converted into the citric acid cycle substrate α-ketoglutarate or could serve as a substrate for glutathione synthesis. We found the citric acid cycle to be significantly deregulated mainly in the G1 and G2 tumors (Fig. 9, Table 3). The observed decreased glutathione level in all EC grades could be due to the overexpression of glutathione peroxidase^64^.

Endometrial cancer is known to be driven by an active hypoxic response^65^—in fact, our NMR results and their multivariate analysis revealed a higher concentration of ascorbate in the G1 tumors than in the G2 and G3 ones (Fig. 6). Similarly, Kuiper et al. showed the concentration of ascorbate to be associated with a tumor grade. It was explained by a reduced capacity of high-grade tumors to accumulate ascorbate as compared to the normal tissue, high hypoxia-inducible factor HIF-1α expression and an increased tumor size^66^. Hypoxia-inducible transcription factors (HIFs) drive angiogenesis and cancer cell growth, thus, the decreased level of ascorbic acid can be related to the more aggressive tumor phenotype. The decreased taurine level in the G3 tumors in comparison to the G1 and G2 ones (Fig. 5) could also be associated with the higher hypoxia in the high grade tumors^67^.

Our final observation of the molecular features of the endometrial cancer concerns dimethyl sulfone (DMSO2), the primary metabolite of dimethyl sulfoxide (DMSO). Interestingly, this metabolite is detected and elevated in the G1 tumors exclusively (Fig. 5). Though DMSO is an agent with a wide spectrum of pharmacological effects, including membrane penetration, anti-inflammatory effects, local analgesia, and weak bacteriostasis, it is important to make clear that according to our knowledge it was not administered to the EC patients included into our study. This chemical is commonly detected in the body fluids (urine, blood, cerebrospinal fluid), brain, skin or eqrwax^68^, derives from dietary sources and is involved in intestinal bacterial metabolism. Although its role in endometrial cancer metabolism remains to be elucidated, it was suggested that dimethyl sulfone might play a beneficial role in the treatment of this cancer^69^.

Although the metabolites resonating in the spectral region from 5.2 to 8.4 ppm were characterized by the minor contribution to the models of the merged aliphatic and aromatic parts of the NMR spectra (Figures S11–S15, Tables S13–S14, Supplementary Materials), the separate multivariate analysis of this region reveals an important information about the metabolic disturbances in endometrial cancer (Figures S16–S19, Tables S15–S16, the Supplementary Materials). Specifically, the decreased ATP in endometrial tumors in comparison to the control tissue (Figure S18c, Supplementary materials) corresponds to the creatine reduction found in the analysis of the region from 0.8 to 4.8 ppm (Fig. 3b,d). The reduced inosine signal in endometrial cancer obtained in our work is in agreement with the findings reported by Altadill et al.^70^. Although the diminished level of the UDP sugars in tumors requires further studies to elucidate the possible biochemical mechanisms, the role of these species in progression of the disease to the advanced stages has already been shown^70^. Our analysis revealed additionally that high glycogen and low inosine levels in tumors are related to the advanced disease stage.

Majority (70–90%) of the patients with endometrial cancer are diagnosed with stages 1–2 of the disease. Low grade tumors detected with early stage are the most common. This is apparent in the clinicopathological characteristics of the studied group in our work. The primary purpose of our work was to find the grade-related differences between the metabolic profiles of endometrial cancer: G1 vs. G2, G2 vs. G3 and G1 vs. G3. To examine the confounding effect of the non-uniform distribution of the disease stages within the grade groups, additional multivariate models were constructed: the G1 (stage 1) tumors were compared with the G2 (stage 1) ones, and the G2 (stage 2 + 3) tumors were compared with G3 (stage 2 + 3) (the Supplementary material). In general, the majority of the changes obtained before stage matching was confirmed using these models. However, elimination of the confounding stage-related effects allows additional disturbances between the different grades to be observed. Interestingly, the metabolome views (Figure S9, Supplementary material) presenting the pathways altered in G1 (stage 1) vs. G2 (stage 1) and G2 (stage 2 + 3) vs. G3 (stage 2 + 3) are similar to those observed before the stage matching (Fig. 9). Stage matching was not possible for the comparison of the G1 to G3 tumors. While all the patients belonging to the G1 group were diagnosed with stage 1, in the G3 group the stage of a cancer at diagnosis was 2 and 3. Inclusion of the high grade tumors diagnosed with stage 1 would be helpful in a better understanding of the relation between the tumors grade/disease stage at the metabolic level. Such analysis is planned in our laboratory.

Although the AUC values, the specificity and sensitivity computed based on the ROC analysis of the cross-validated predicted Y values of the ^1^H NMR OPLS-DA models indicate an excellent performance of the technique in differentiation between the G1 vs. G2, G1 vs. G3 and G2 vs. G3 tumors (Figure S1, Supplementary materials), the external validation of the obtained results is required. The obtained findings should also be confirmed in the targeted analysis. Our results obtained for the post-surgical tissue samples encourage to examine the utility of the HR MAS NMR technique or liquid state NMR methods (analysis of extracted metabolites) also for the pre-operative metabolic profiling of tissue in the EC diagnostics and research studies. There are some promising results: the metabolites extracted from the endometrial tissue obtained by dilation and curettage were studied using NMR metabolomics^71^. Some tumor metabolites find their way into the bloodstream, undergo chemical modification and are excreted in urine. Blood-based metabolites are easily accessible but their diagnostic potential is limited by dilution and thus the low yield of cancer-derived metabolites in blood. Further studies exploring these possibilities are urgently needed.

One of our future aims is also to compare the results presented in our work with the results obtained from absolute quantitation. Although NMR spectroscopy is a quantitative technique, evaluation of the absolute metabolite concentrations in the morphologically heterogeneous tumor tissue is not trivial^24,72^. Histopathological verification of the samples previously examined using HR MAS NMR technique and evaluation of the percentage content of various components of the tumor microenvironment (fibrosis, inflammation, necrosis, etc.) would be necessary to better understand the discrepancies between these two approaches.

## Supplementary Information

Supplementary Information.

## Data Availability

The datasets generated during and/or analysed during the current study are available from the corresponding author on reasonable request.
