# Supplementary material for: Grading of endometrial cancer using 1H HR-MAS NMR-based metabolomics
Source: Sci Rep. 2021 Sep 13;11:18160. doi: 10.1038/s41598-021-97505-y (PMC8438077; doi:10.1038/s41598-021-97505-y)
Supplement: Supplementary file 1 — Supplementary Information. [file 41598_2021_97505_MOESM1_ESM.pdf]

SUPPLEMENTARY MATERIALS

Grading of endometrial cancer using ^{1}H HR MAS NMR-based metabolomics

Agnieszka Skorupa[1], Michał Poński[2], Mateusz Ciszek[1], Bartosz Cichoń[2], Mateusz Klimek[2], Andrzej Witek[2], Sławomir Pakuło[3], Łukasz Boguszewicz[1], Maria Sokół[1]

[1] Department of Medical Physics, Maria Skłodowska-Curie National Research Institute of Oncology, Gliwice Branch, 44-102, Gliwice, Poland.

[2] Department of Gynaecology and Obstretrics, Medical University of Silesia in Katowice, Medyków 14, 40-752 Katowice, Poland

[3] Tumor Pathology Department, Maria Skłodowska-Curie National Research Institute of Oncology, Gliwice Branch, 44-102, Gliwice, Poland.

*Corresponding author: agnieszka.skorupa@io.gliwice.pl

1. Quality Control

The QC procedure contains three elements: the requirements of the tissue specimen preparation, the QC of the NMR protocol and the QC of the multivariate data analysis. As in all bioanalytical approaches, a clean and reliable sample preparation strategy is a significant component in designing metabolomics (or -omics, in general) studies. Our tissue sample preparation procedures were defined and rigorously followed. To ensure robust and accurate quantification of the potential biomarkers by NMR, each step of the analytical protocol was carefully performed and evaluated. One of our QC approaches is to keep the post-processing procedure simple and to perform the multivariate analyses using raw or minimally processed data which does not rely, for example, on high-level processing.

The detailed information concerning all three stages of the QC procedure are as follows:

Sample preparation

To minimize the sample degradation processes the time between the tissue resection and freezing was kept as short as possible (below 5 minutes). The cutting of the sample for the NMR measurements was performed on a metal block cooled with liquid nitrogen to keep the sample frozen. The NMR measurements were performed at 4°C.

NMR quality control procedures

The quality control procedures included:

- Magic angle adjustment using bromide potassium sample (performed once a month, or after a probe change).
- Temperature calibration using 4% methanol dissolved in d3-methanol sample (done once a month, or after a probe change).
- Manual shimming the probe using a sample of 3% CHCl3 in Acetone-d6 (every day)

The acquisition of each HR MAS NMR spectrum was proceeded by: manual tuning and matching of the probe, locking the Bo filed using D20, manual shimming using the added formate signal (FWHM < 1.5 Hz), manual adjustment of the pulse length and manual determination of the transmitter frequency offset (O1) for optimal water suppression.

In order to make the spectra comparable to each other the probabilistic quotient normalization was used.

OPLS-DA modeling details

Each OPLS-DA model was described using the number of the model components, the fractions of the X and Y variation explained by the predictive and orthogonal components (R2X and R2Y), the fractions of the Y variation predicted by the models (Q2) and the p-values from the CV-Anova test. The combination of VIP and p(corr)[1] was used for variable selection.

2. The chemical shift assignments in the spectral region from 0.8 to 4.8 ppm

**Metabolite**	**Chemical shift [ppm]/multiplicity**
Fatty acids	0.89 (t)
Isoleucine	0.93 (t)
Leucine	0.94 (d)
Leucine	0.96 (d)
Valine	0.98 (d)
Isoleucine	1.00 (d)
valine	1.04 (d)
3-hydroxybutyrate	1.20 (d)
Fatty acids	1.29 (broad)
Lactate	1.33 (d)
Alanine	1.47 (d)
Lysine	1.72 (m)
Acetate	1.91 (s)
N-acetyl compound	2.02 (s)
Glutamate	2.05 (m)
UDP-sugars	2.08 (s)
Glutamate	2.11 (m)
Glutamine	2.13 (m)
Methionine	2.13 (s)
Glutathione	2.17 (m)
3-hydroxybutyrate	2.31 (dd)
Glutamate	2.35 (dt)
Succinate	2.41 (s)
3-hydroxybutyrate	2.41 (dd)
Glutamine	2.45 (m)
Glutathione	2.55 (m)
Methionine	2.63 (t)
Hypotaurine	2.65 (t)
Aspartate	2.68 (dd)
Aspartate	2.80 (dd)
Glutathione	2.97 (m)
Lysine	3.01 (t)
Creatine	3.03 (s)
Creatinine	3.05 (s)
Ethanolamine	3.13 (t)
Dimethyl sulfone	3.15 (s)
Choline	3.20 (s)
Phosphocholine	3.22 (s)
Glycerophosphocholine	3.23 (s)
Phosphoethanolamine	3.23 (t)
Taurine	3.25 (t)
Betaine	3.27 (s)
Myo-inositol	3.27 (t)
Hypotaurine	3.34 (t)
Scyllo-inositol	3.34 (s)

Taurine	3.43 (t)
Myo-inositol	3.53 (dd)
Glycine	3.56 (s)
Myo-inositol	3.62 (t)
Glycerophosphocholine	3.70 (m)
Amino acid residues	3.75- 4.00
Serine	3.83 (dd)
Ethanolamine	3.83 (t)
Betaine	3.92 (s)
Creatine	3.93 (s)
Serine	3.97 (m)
Phosphoethanolamine	4.00 (m)
Myo-inositol	4.06 (t)
Lactate	4.13 (q)
Phosphocholine	4.16 (m)
Glycerophosphocholine	4.33 (m)
Ascorbate	4.53 (d)
Glutathione	4.58 (t)
β-Glucose	4.65 (d)

Table S1. NMR assignment for the metabolites detected in endometrial cancer and control tissue.

s - singlet, d – doublet, dd – doublet of doublets, t- triplet, q – quartet, m – multiplet

3. Data supplemetary to OPLS-DA models 4-9

**Model**	**CV-Anova p-value**	**Number of components**	**R2X [%]**	**R2Y [%]**	**Q2 [%]**	**Intercepts obtained from the permutation test**	
						**R2Y [%]**	**Q2Y[%]**
G1 tumors vs. Control tissue (model 4)	5.14407e-11	1 predictive	31.5	96.4	93.5	31.7	-46.4
		1 orthogonal	30.3				
G2 tumors vs Control tissue (model 5)	8.04113e-20	1 predictive	19.4	93.9	91.5	22.5	-41.3
		1 orthogonal	30.1				
G3 tumors vs Control tissue (model 6)	2.29103e-16	1 predictive	39.5	99.1	98.2	36.9	-58.0
		1 orthogonal	24.4				

Table S2. OPLS-DA models (4-6) diagnostics. Number of the OPLS-DA model components, the p-values obtained from the CV Anova test, the fractions of the total X and Y variation explained by the model (R2X, R2Y), the fractions of the total Y variation that can be predicted by the model (Q2), the intercepts values obtained from the permutation tests representing the values of R2Y and Q2 of the purely random models.

Metabolite		Chemical shift [ppm]	Grade 1 endometrial cancer vs. Control tissue (OPLS-DA model 4)			Grade 2 endometrial cancer vs. Control tissue (OPLS-DA model 5)			Grade 3 endometrial cancer vs. Control tissue (OPLS-DA model 6)		
			P(corr)[1]	VIP	Fold change (p value)	P(corr)[1]	VIP	Fold change (p value)	P(corr)[1]	VIP	Fold change (p value)
BCAA	Leucine	0.96	-0.46	1.45	1.13 (0.053240)	-0.47	1.29	1.33 (0.000050)	-0.69	1.48	1.55 (0.000021)
	Isoleucine	1.02	-0.55	0.73	1.10 (0.126461)	-0.48	0.57	1.35 (0.000203)	-0.66	0.79	1.52 (0.000010)
	Valine	1.04	-0.50	1.03	1.19 (0.043522)	-0.48	0.73	2.29 (0.000377)	-0.56	1.04	1.32 (0.000733)
Lactate		1.32	-0.60	7.69	1.20 (0.354899)	-0.38	7.23	1.19 (0.013650)	-0.72	7.37	1.32 (0.002172)
		4.13	-0.64	3.31	1.24 (0.307902)	-0.41	3.73	1.31 (0.056636)	-0.74	3.81	1.47 (0.020741)
Alanine		1.47	-0.39	2.18	0.99 (0.733897)	-0.36	1.98	1.48 (0.006414)	-0.75	2.15	1.48 (0.005191)
Lysine		1.72	-0.64	1.13	1.48 (0.000220)	-0.53	0.87	1.48 (0.000048)	-0.64	0.91	1.38 (0.000050)
N-acetyl group		2.02	-0.60	1.44	1.38 (0.004796)	-0.39	1.27	1.54 (0.000980)	-0.87	1.60	2.41 (0.000000)
Hypotaurine		2.65	-0.83	1.08	1.95 (0.000017)	-0.67	0.92	1.93 (0.000021)	-0.66	0.71	1.74 (0.002194)
Dimethyl sulfone		3.15	-0.69	3.72	10.27 (0.000000)	Not detected			Not detected		
Phosphocholine		3.22	-0.51	3.03	Direct integration: 1.14 (0.173463)<br><br>Line fitting:	-0.33	4.28	Direct integration: 1.05 (0.664758)<br><br>Line fitting: 1.42	-0.37	3.91	Direct integration: 1.31 (0.119887)<br><br>Line fitting: 1.13

				1.57 (0.022826)			(0.010005)			(1.000000)
Glycerophosphocholine	3.23	0.59	2.21	Direct integration: 0.85 (1.000000)<br><br>Line fitting: 0.49 (0.040526)	0.14	2.10	Direct integration: 0.82 (1.000000)<br><br>Line fitting: 0.49 (0.019664)	-0.32	5.04	Direct integration: 1.05 (1.000000)<br><br>Line fitting: 1.46 (1.00000)
	3.70	0.67	0.92	nd	0.29	0.58	nd	-0.42	1.55	nd
	4.33	0.40	0.38	0.95 (1.000000)	0.30	0.25	0.94 (0.829776)	-0.49	1.32	1.88 (1.000000)
Choline	3.20	-0.94	5.82	3.11 (0.000000)	-0.76	4.66	2.66 (0.000006)	-0.48	2.44	1.48 (0.155616)
Taurine	3.43	-0.85	5.31	2.02 (0.000097)	-0.64	4.41	1.71 (0.000130)	-0.20	1.94	1.13 (1.000000)
Glycine	3.56	-0.55	4.03	1.15 (0.062700)	-0.31	2.08	1.15 (0.176545)	-0.55	3.11	1.15 (0.071890)
Serine	3.97	-0.92	3.10	3.05 (0.000000)	-0.71	2.41	1.93 (0.000955)	-0.79	1.59	1.62 (0.035124)
Glutamate	2.35	0.81	1.52	0.79 (0.000085)	0.46	1.05	0.89 (0.007355)	0.52	1.03	0.83 (0.001385)
Glutamine	2.45	0.63	0.78	0.73 (0.066943)	0.40	0.58	0.79 (0.064513)	0.46	0.45	0.80 (0.061527)
Glutathione	2.55	0.79	1.18	nd	0.68	1.12	nd	0.82	1.08	nd
	2.96	0.66	1.01	nd	0.60	1.06	nd	0.87	1.09	nd
	4.58	0.85	1.15	0.65 (0.041504)	0.71	1.03	0.62 (0.000000)	0.79	1.00	0.55 (0.000083)
Creatine	3.03	0.94	6.26	0.46 (0.000258)	0.93	6.91	0.37 (0.000001)	0.96	5.65	0.31 (0.012408)
	3.93	0.94	4.18	0.51 (0.014592)	0.92	4.50	0.46 (0.000010)	0.96	3.53	0.52 (0.000001)
Scyllo-inositol	3.34	0.82	3.41	0.71	0.83	3.80	0.44	0.98	3.83	0.29

				(0.028999)			(0.000128)			(0.000000)
Ascorbate	4.53	0.76	1.89	0.54 (0.002104)	0.93	2.61	0.31 (0.000424)	0.92	1.99	0.32 (0.000006)
3-hydroxybutyrate	1.19	-0.41	0.46	1.43 (0.094604)	-0.43	1.29	1.70 (0.000031)	-0.66	1.50	3.37 (0.000000)
Creatinine	3.05	0.82	2.03	0.33 (0.000089)	0.82	2.14	0.34 (0.000002)	0.79	1.59	0.48 (0.001755)
Myo-inositol	3.62	0.06	3.44	nd	0.57	4.66	nd	0.98	5.44	nd
	3.55	0.06	3.19	nd	0.56	4.43	nd	0.98	5.00	nd
	4.06	0.06	2.50	1.32 (1.000000)	0.53	3.42	0.61 (0.155643)	0.98	4.03	0.30 (0.000041)
Ethanolamine	3.13	-0.81	0.93	1.98 (<0.000001)	-0.50	0.70	1.52 (0.002543)	-0.74	0.79	1.85 (0.000035)
Phosphoethanolamine	4.00	0.94	2.64	0.46 (0.000007)	0.74	2.31	0.65 (0.000043)	0.55	1.56	0.68 (0.008570)
Betaine	3.92	Not detected			Not detected			-0.45	2.19	1.93 (0.881833)

nd – not determined

Table S3. Metabolites contributing to the differentiation between the EC malignancy grades (G1-G3) and the control tissue. The positive p(corr)[1] values correspond to the higher metabolite levels in the normal endometrium compared to the cancerous tissue.

VIP – Variable Importance at Projection, p(corr)[1] – the loadings scaled as the correlations for the predictive components of the OPLS-DA models. The fold changes were computed as a ratio of the median metabolite level in the cancerous tissue to the median metabolite level in the normal endometrium, the p-values were obtained from the multiple comparisons of the mean ranks following the Kruskal-Wallis test.

Model	CV-Anova p-value	Number of components	R2X [%]	R2Y [%]	Q2 [%]	Intercepts obtained from the permutation test	
						R2Y [%]	Q2 Y[%]
G1 vs G2 (model 7)	5.59604e-06	1 predictive	8.03	89.1	61.4	27.3	-37.9
		3 orthogonal	51.5				
G2 vs G3 (model 8)	1.15218e-08	1 predictive	28.0	92.9	84.6	41.0	-58.9
		2 orthogonal	37.6				
G1 vs G3 (model 9)	3.05708e-12	1 predictive	15.3	81.0	72.9	27.3	-37.9
		1 orthogonal	24.4				

Table S4. OPLS-DA models (7-9) diagnostics. Number of the OPLS-DA model components, the p-values obtained from the CV Anova test, the fractions of the total X and Y variation explained by the model (R2X, R2Y), the fractions of the total Y variation that can be predicted by the model (Q2), the intercepts values obtained from the permutation tests representing the values of R2Y and Q2 of the purely random models.

Metabolite	Chemical shift [ppm]	P(corr)[1]	VIP	Fold change (p-value)	AUC (95% confidence band)
Dimethyl sulfone	3.15	-0.78	5.41	8.43 (0.000022)	0.948 (0.863-0.996)
Myo-inositol	3.55	-0.48	5.96	nd	nd
	3.62	-0.49	6.44	nd	nd
	4.06	-0.48	4.66	2.17 (0.164266)	0.719 (0.542-0.896)
Serine	3.97	-0.48	2.33	1.58 (0.008616)	0.784 (0.615-0.939)
Ascorbate	4.52	-0.56	1.45	1.73 (0.050366)	0.792 (0.564-0.959)

nd – not determined

Table S5. Metabolites contributing to the differentiation between the G1 and G2 endometrial cancer (model 7). The positive p(corr)[1] values indicate the higher metabolite level in G2 compared to G1. VIP – Variable Importance at Projection, p(corr)[1] – the loadings scaled as the correlations for the predictive component of the OPLS-DA models, AUC – area under the ROC curve. The fold changes were computed as a ratio of the median metabolite level in the G1 tumor to the median metabolite level in the G2 tumor; the p-values were obtained from the multiple comparisons of the mean ranks following Kruskal-Wallis test.

Metabolite	Chemical shift [ppm]	P(corr)[1]	VIP	Fold change (p value)	AUC (95% confidence band)
Glycerophosphocholine	3.23	-0.48	5.79	Direct integration: 0.78 (0.739355)<br><br>Line fitting: 0.33 (0.020227)	Line fitting: 0.779 (0.61-0.904)
	3.70	-0.69	2.30	nd	nd
	4.33	-0.72	1.83	0.50 (0.003101)	nd
Betaine	3.92	-0.68	3.00	0.42 (0.013788)	0.738 (0.581-0.876)
	3.27	-0.51	3.75	nd	nd
Choline	3.20	0.63	4.04	1.80 (0.015312)	0.849 (0.728-0.944)
Scyllo-inositol	3.34	0.64	2.44	1.52 (0.006413)	0.918 (0.827-0.988)
Taurine	3.42	0.60	4.42	1.52 (0.000057)	0.923 (0.839-0.98)
Myo-inositol	3.55	0.58	4.68	nd	nd
	3.62	0.62	5.17	nd	nd
	4.06	0.60	3.93	2.03 (0.005540)	0.889 (0.788-0.963)

nd – not determined

Table S6. Metabolites contributing to the differentiation between the G2 and G3 endometrial cancers (model 8). The positive p(corr)[1] values indicate the higher metabolite levels in G2 compared to G3. VIP – Variable Importance at Projection, p(corr)[1] – the loadings scaled as the correlations for the predictive component of the OPLS-DA models, AUC – area under the ROC curve. The fold changes were computed as a ratio of the median metabolite level in the G2 tumors to the median metabolite level in the G3 tumors; the p-values were obtained from the multiple comparisons of the mean ranks following Kruskal - Wallis test.

Metabolite	Chemical shift [ppm]	P(corr)[1]	VIP	Fold change (p-value)	AUC (95% confidence band)
3-hydroxybutyrate	1.19	-0.64	1.50	0.42 (0.008770)	0.929 (0.813-1)
Glycerophosphocholine	3.23	-0.41	5.59	Direct integration: 0.81 (0.619996)<br><br>Line fitting: 0.33 (0.059405)	Line fitting: 0.794 (0.622-0.916)
	3.70	-0.58	1.70	nd	nd
	4.33	-0.54	1.42	0.50 (0.038296)	nd
Betaine	3.92	-0.49	2.31	0.42 (0.04036)	0.735 (0.544-0.906)
	3.27	-0.28	3.15	nd	nd
Ascorbate	4.52	0.52	1.01	1.66 (0.567351)	0.693 (0.477-0.866)
Serine	3.97	0.79	2.10	1.89 (0.008616)	0.924 (0.722-1)
Lactate	4.13	-0.52	1.42	0.84 (0.475966)	0.807 (0.597-0.933)
Myo-inositol	3.55	0.74	5.43	nd	nd
	3.62	0.74	5.90	nd	nd
	4.06	0.75	4.36	4.41 (0.000016)	0.899 (0.767-1)
Taurine	3.42	0.81	4.77	1.79 (0.000082)	0.95 (0.83-1)
Scyllo-inositol	3.34	0.77	2.72	2.45 (0.001187)	0.874 (0.716-1)
Choline	3.20	0.78	4.25	2.11 (0.000141)	0.962 (0.863-1)
Dimethyl sulfone	3.15	0.77	3.48	7.16 (0.007290)	0.937 (0.798-1)
Creatine	3.03	0.55	2.21	1.47 (1.000000)	0.79 (0.576-0.95)
Phosphoethanolamine	4.00	-0.49	1.45	0.68 (0.209036)	0.813 (0.639-0.948)

nd – not determined

Table S7. Metabolites contributing to the differentiation between the G1 and G3 endometrial cancers (model 9). The positive p(corr)[1] values correspond to the higher metabolite levels in the G1 tumor than in the G3 ones. VIP – Variable Importance at Projection, p(corr)[1] – the loadings scaled as the correlations for the predictive component of the OPLS-DA model, AUC – area under the ROC curve. The fold changes were computed as a ratio of a median metabolite level in the G1 tumors to the median

metabolite level in the G3 tumors; the p-values were obtained from the multiple comparisons of the mean ranks following Kruskal - Wallis test.

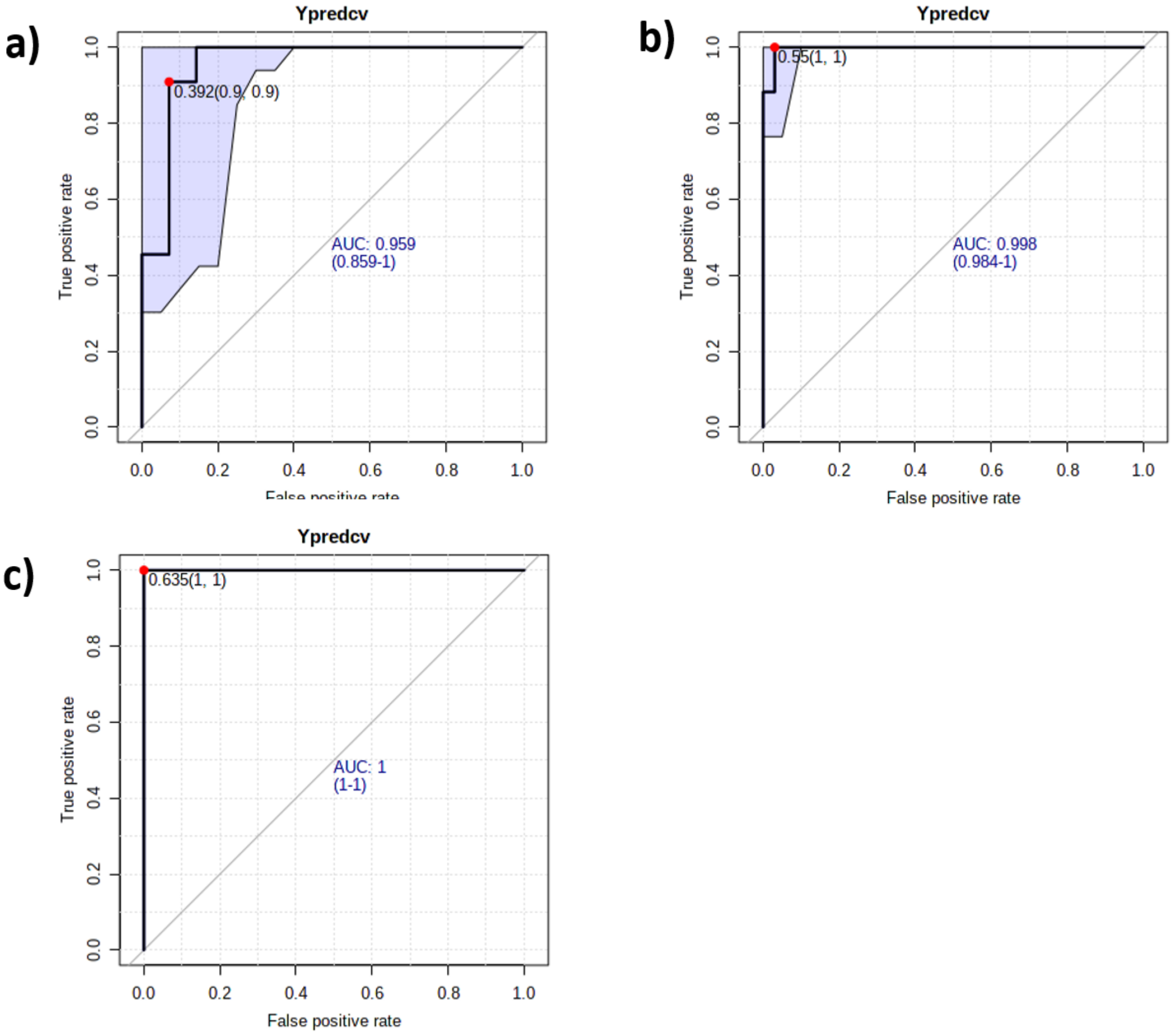

Figure S1. ROC curves for classification by OPLS-DA models based on seven-fold cross-validation predicted Y-scores: (a) G1 vs. G2 tumors (model 7), (b) G2 vs. G3 tumors (model 8), (c) G1 vs. G3 tumors (model 9). The image was created using Metaboanalyst 4.0 software (https://www.metaboanalyst.ca).

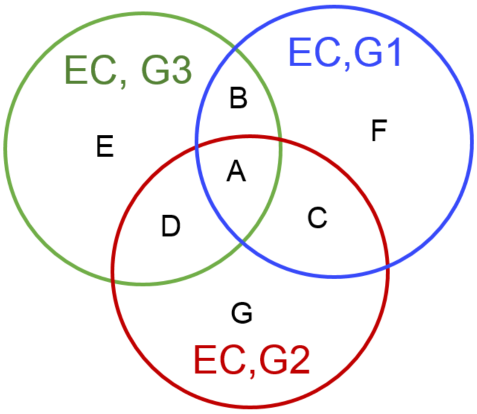

A: ascorbate, creatine, creatinine, scyllo-inozytol, choline, hypotaurine, phosphoethanolamine, serine, ethanolamine, glutathione, lysine, glutamate, isoleucine, leucine, valine

B: glycine, lactate, N-acetyl compound

C: taurine

D: myo-inositol

E: alanine, 3-hydroxybutyrate, betaine

F: dimethyl sulfone, phosphocholine, glycerophosphocholine, glutamine

G: -

Figure S2. Venn diagram showing the number of differentiating metabolites (common and unique) to each tumor grade in reference to control tissue according to the OPLS-DA models 4-6.

5. Multivariate models S1-S4

Although the direct analysis of the influence of the disease advancement on the metabolic profiles within each pathomorphological grade group is not possible in our work, several multivariate models were constructed to shed some light on this problem (Figure S3). Two separate OPLS-DA models distinguishing the G2 (stage 1) tumors from the control tissue (OPLS-DA model S1) and the G2 (stage 2+3) tumors from this tissue (OPLS-DA model S2) were compared to each other by means of the SUS plot. To examine the confounding effect of the disease stage on the metabolic differences between G1 and G2 endometrial cancer, the OPLS-DA model S3 was built based on the G1 and G2 (stage 1) tumors. The patients with the G2 and G3 tumors characterized by the more advanced disease stage (stages 2+3) were included in the development of the OPLS-DA model S4.

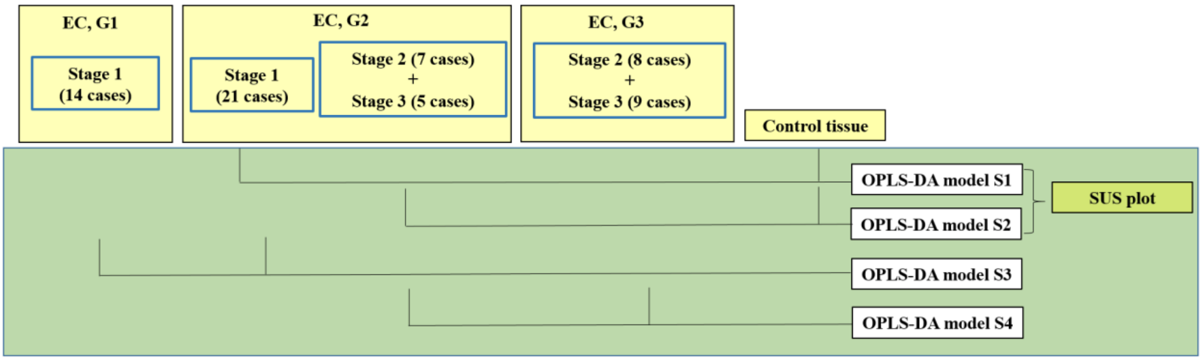

Figure S3. The scheme presenting the constructed multivariate models (S1-S4).

The number of the model components, the fractions of the X and Y variation explained by the predictive and orthogonal components (R2X and R2Y), the fractions of the Y variation predicted by the OPLS-DA models S1-S4 (Q2), the p-values from CV-Anova test and the results from the permutation testing are presented in Table S8.

Model	CV-Anova P value	Number of components	R^2X [%]	R^2Y [%]	Q2 [%]	Intercepts obtained from the permutation test	
						R^2Y [%]	Q^2 Y[%]
G2 (stage 1) tumors vs. Control tissue (model S1)	2.79797e-16	1 predictive	21.8	96.3	94.8	26.1	-44.2
		1 orthogonal	33.8				
G2 (stage 2+3) tumors vs. Control tissue (model S2)	3.72029e-09	1 predictive	44.2	96.9	92.1	30.2	-46.0
		1 orthogonal	18.1				
G1 (stage 1) tumors vs. G2 (stage 1) tumors (model S3)	0.000970692	1 predictive	6.78	89.9	59.9	60.9	-79.6
		3 orthogonal	61.5				
G2 (stage 2+3) tumors vs. G3 (stage 2+3) tumors (model S4)	1.16583e-08	1 predicitve	14.9	92.7	82.1	42.8	-46.4
		2 orthogonal	16.4				

Table S8. OPLS-DA models (S1-S4) diagnostics. Number of the components in the models, the p-values obtained from CV Anova test, the fractions of the total X and Y variation explained by the model (R2X, R2Y), the fractions of the total Y variation that can be predicted by the model (Q2), the intercepts values obtained from the permutation tests representing the values of R2Y and Q2 of the purely random models.

OPLS-DA models S1 and S2

The scores plots obtained from OPLS-DA models S1 and S2 differentiating G2 (stage 1) and G2 (stages 2+3) tumors from the control tissue are shown in Figures S4-S5. Figure S6 presents the SUS plot comparing these models. The p(corr)[1] and VIP values for the most important metabolites obtained from these models are listed in Table S9. The common features of the G2 (stage 1) and G2 (stage 2+3) tumors in reference to the control tissue include: higher 3-hydroxybutyrate, N-acetyl compound, isoleucine, leucine, valine, lysine, taurine, serine, hypotaurine, choline and ethanolamine and decreased glutamate, creatinine, glutathione, scyllo-inositol, creatine and ascorbate. Increased lactate, alanine and phosphocholine and decreased glucose, myo-inositol, acetate and glutamine were found to be characteristic for the G2 (grade 2+3) group in relation to the non-transformed tissue [not observed in the G2 (stage 1) tumor group], while the lower succinate was distinctly observed in the G2 (grade 1) tumors group in relation to the normal tissue.

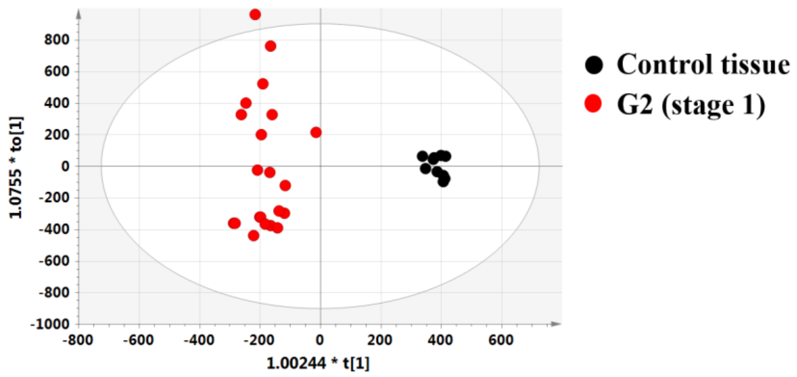

Figure S4. Scores plot obtained from the OPLS-DA model S1 differentiating G2 (stage 1) tumors from the normal endometrium. The image was created using SIMCA-P 15.0 software package (https://www.sartorius.com).

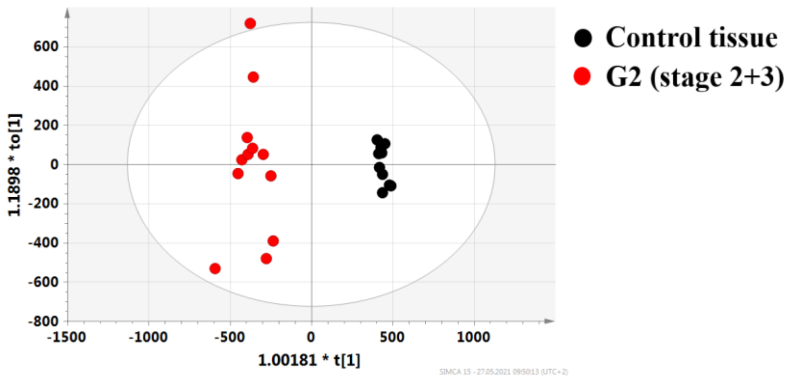

Figure S5. Scores plot obtained from the OPLS-DA model S2 differentiating G2 (stages 2+3) tumors from the normal endometrium. The image was created using SIMCA-P 15.0 software package (https://www.sartorius.com).

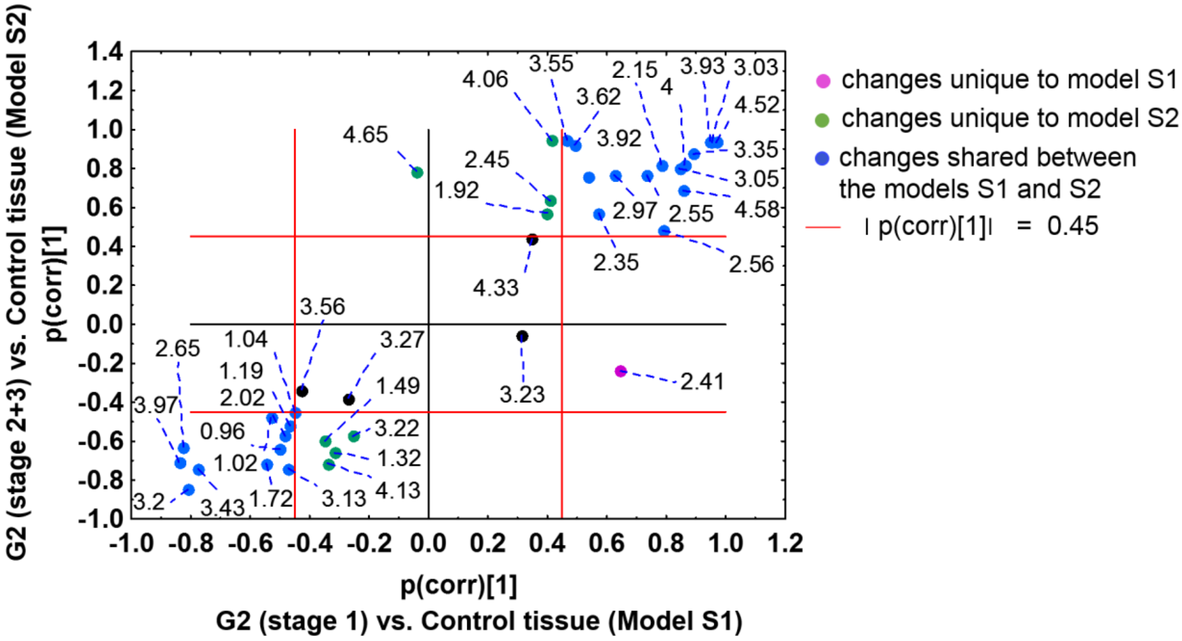

Figure S6. SUS plot comparing OPLS-DA models S1 and S2. The image was created using Statistica 12.5 software (www.statsoft.pl) based on data exported from SIMCA-P 15.0 software package (https://www.sartorius.com).

			G2 tumors (stage 1) vs. control tissue (OPLS-DA model S1)		G2 tumors (stage 2+3) vs. control tissue (OPLS-DA model S2)	
**Changes**	**Metabolite**	**Chemical shift [ppm]**	**P(corr) [1]**	**VIP**	**P(corr)[1]**	**VIP**
Changes common to OPLS-DA models S1 and S2	Leucine	0.96	-0.50	1.40	-0.64	1.24
	Isoleucine	1.02	-0.53	0.67	-0.48	0.51
	Valine	1.04	-0.45	0.85	-0.45	0.72
	Lysine	1.72	-0.55	0.93	-0.71	0.80
	N-acetyl group	2.02	-0.48	1.00	-0.57	1.69
	Hypotaurine	2.65	-0.82	1.07	-0.63	0.70
	Choline	3.20	-0.81	5.21	-0.85	3.59
	Taurine	3.43	-0.77	4.33	-0.74	4.52
	Serine	3.97	-0.84	2.84	-0.71	1.49
	Glutamate	2.35	0.57	1.23	0.57	0.99
	Glutathione	2.55	0.73	1.24	0.77	0.95
		2.97	0.63	1.11	0.77	0.96
		4.58	0.86	1.23	0.69	0.86
	Creatine	3.03	0.95	7.36	0.94	5.60
		3.93	0.95	4.77	0.94	3.76
	Scyllo-inositol	3.34	0.89	4.13	0.87	3.23
	Ascorbate	4.53	0.97	2.84	0.93	2.13
	3-hydroxybutyrate	1.19	-0.47	1.35	-0.52	1.30
	Creatinine	3.05	0.95	7.37	0.93	5.60
	Ethanolamine	3.13	-0.47	0.66	-0.74	0.71
	Phosphoethanolamine	4.0	0.86	2.80	0.82	1.55
Changes specific to OPLS-DA model S2	Glucose	4.64	-0.04	0.78	0.79	1.10
	Lactate	1.32	-0.32	5.97	-0.66	8.12
		4.13	-0.34	3.07	-0.71	4.13
	Alanine	1.47	-0.35	2.12	-0.60	1.98
	Phosphocholine	3.22	-0.25	4.21	-0.57	4.84
	Glutamine	2.45	0.41	0.61	0.64	0.66
	Myo-inositol	3.62	0.49	4.04	0.92	4.88

		3.55	0.47	3.72	0.95	4.72
		4.06	0.41	2.88	0.94	3.68
	Acetate	1.91	0.40	0.82	0.57	0.91
Changes specific to OPLS-DA model S1	Succinate	2.41	0.65	1.50	-0.24	0.41

Table S9. Metabolites contributing to the differentiation between the G2 (stage 1) tumors and the normal endometrium (OPLS-DA model S1) and the G2 stage (2+3) tumors and the normal endometrium (OPLS-DA model S2). The positive p(corr)[1] values indicate the higher metabolite levels in the normal tissue in comparison to the tumorous one. VIP – Variable Importance at Projection, p(corr)[1] – the loadings scaled as the correlations for the predictive component of the OPLS-DA models.

OPLS-DA model S3

The scores plot obtained from OPLS-DA model S3 differentiating G1 (stage 1) from G2 (stage 1) tumors is presented in Figure S7, while the pcorr(1) and VIP values for the most important metabolites obtained from these models are shown in Table S10. The G2 (stage 1) tumors are characterized by the lower succinate, serine, dimethyl sulfone, ascorbate and taurine than the G1 (stage 1) tumors. These metabolites are characterized by AUC > 0.7.

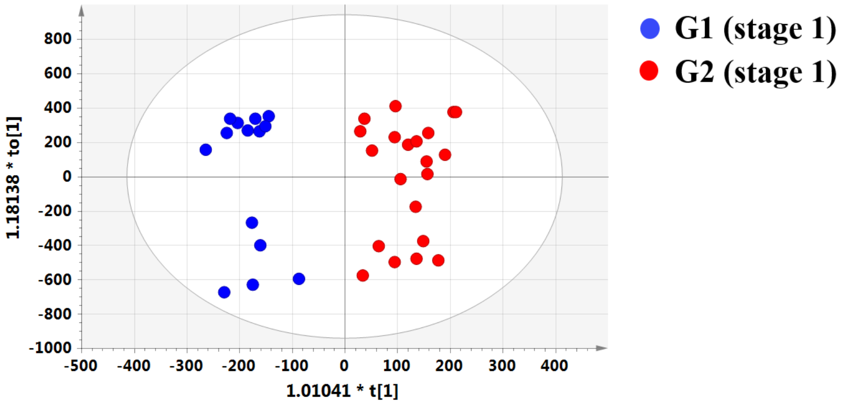

Figure S7. Scores plot obtained from the OPLS-DA model S3 differentiating G1 (stage 1) from G2 (stage 1) tumors. The image was created using SIMCA-P 15.0 software package (https://www.sartorius.com).

Metabolite	Chemical shift [ppm]	P(corr)[1]	VIP	AUC (95% confidence interval)
Succinate	2.41	-0.55	2.48	0.789 (0.59-0.944)
Serine	3.97	-0.49	2.38	0.733 (0.52-0.901)
Dimethyl sulfone	3.15	-0.77	6.24	0.959 (0.871-1)
Ascorbate	4.52	-0.58	2.07	0.803 (0.588-0.99)
Taurine	3.43	-0.45	4.44	0.724 (0.527-0.912)

Table S10. Metabolites contributing to the differentiation between the grade 1 (stage 1) and grade 2 (stage 1) endometrial cancer (model S3). The positive p(corr)[1] values indicate the higher metabolite levels in the grade 2 (stage 1) cancer compared to the grade 1 (stage 1) cancer. VIP – Variable Importance at Projection, p(corr)[1] – the loadings scaled as correlations for the predictive component of the OPLS-DA models, AUC – area under the ROC curve.

OPLS-DA model S4

The scores plot obtained from the OPLS-DA model S4 differentiating the G2 (stage 2+3) from G3 (stage 2+3) tumors is presented in Figure S8, while the pcorr(1) and VIP values for the most important metabolites obtained from these models are shown in Table S11. Higher choline, scyllo-inositol, taurine, myo-inositol, creatine and succinate and lower betaine, ascorbate and glucose were observed in the G2 (stage 2+3) tumors than in the G3 (stage 2+3) tumors. However, creatine and ascorbate are characterized by AUC < 0.7. Although the ׀p(corr)[1]׀ value for the glycerophosphocholine signal at 3.23 ppm is below 0.45, the AUC obtained from ROC analysis of the integrated signal of this metabolite (derived from line fitting) is above 0.75. The p(corr)[1] values found for the remaining signals corresponding to glycerophosphocholine (3.70 ppm, 4.33) indicate the increased level of this metabolite in G3 (stage 2+3) tumors than in G2(stage 2+3) ones.

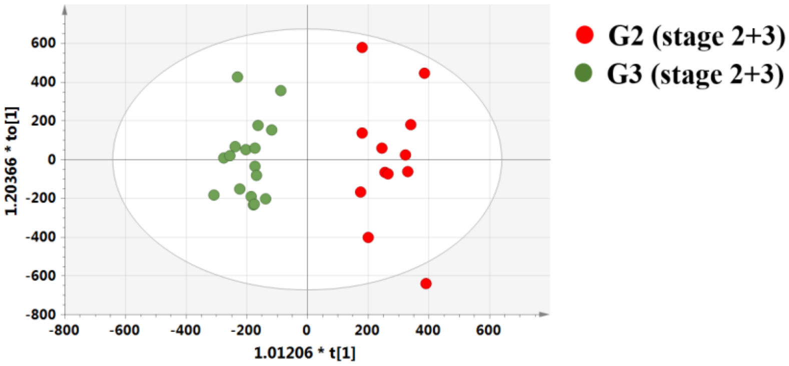

Figure S8. Scores plot obtained from the OPLS-DA model S4 differentiating the G2 (stage 2+3) from G3 (stage 2+3) tumors. The image was created using SIMCA-P 15.0 software package (https://www.sartorius.com).

**Metabolite**	**Chemical shift [ppm]**	**P(corr)[1]**	**VIP**	**AUC (95% confidence interval)**
Glycerophosphocholine	3.23	-0.34	4.82	Line fitting: 0.765 (0.569-0.897)
	3.70	-0.65	2.34	nd
	4.33	-0.63	1.94	nd
Betaine	3.92	-0.59	3.12	0.897 (0.762-0.993)
Choline	3.20	0.49	3.58	0.853 (0.676-0.963)
Scyllo-inositol	3.34	0.53	2.18	0.838 (0.637-1)
Taurine	3.43	0.63	6.05	0.873 (0.706-1)
Myo-inositol	3.55	0.31	2.15	nd
	3.62	0.46	2.92	nd
	4.06	0.46	2.08	0.811 (0.576-0.973)
Glucose	4.64	-0.47	1.21	0.809 (0.591-0.983)
Succinate	2.41	0.52	1.56	0.745 (0.531-0.912)
Creatine	3.03	0.48	1.08	0.598 (0.38-0.824)
Ascorbate	4.52	-0.47	1.18	0.598 (0.37-0.821)

nd – not determined

Table S11. Metabolites contributing to the differentiation between the G2 (stage 2+3) and G3 (stage 2+3) endometrial cancer (model S4). The positive p(corr)[1] values indicate the higher metabolite levels in the G2 (stage 2+3) cancer compared to the G3 (stage 2+3) cancer. VIP – Variable Importance at Projection, p(corr)[1] – the loadings scaled as correlations for the predictive component of the OPLS-DA models, AUC – area under the ROC curve.

Pathway analysis

Metabolome views obtained from the analysis of the metabolic differences between G1 (stage 1) vs. G2 (stage 1) tumors and between G2 (stage 2+3) and G3 (stage 2+3) tumors are presented in Figure S9.

Interestingly, similar metabolic pathways were found to be disturbed before (Figure 9) and after stage matching (Figure S9) for the comparisons of G1 to G2 tumors and G2 to G3 tumors.

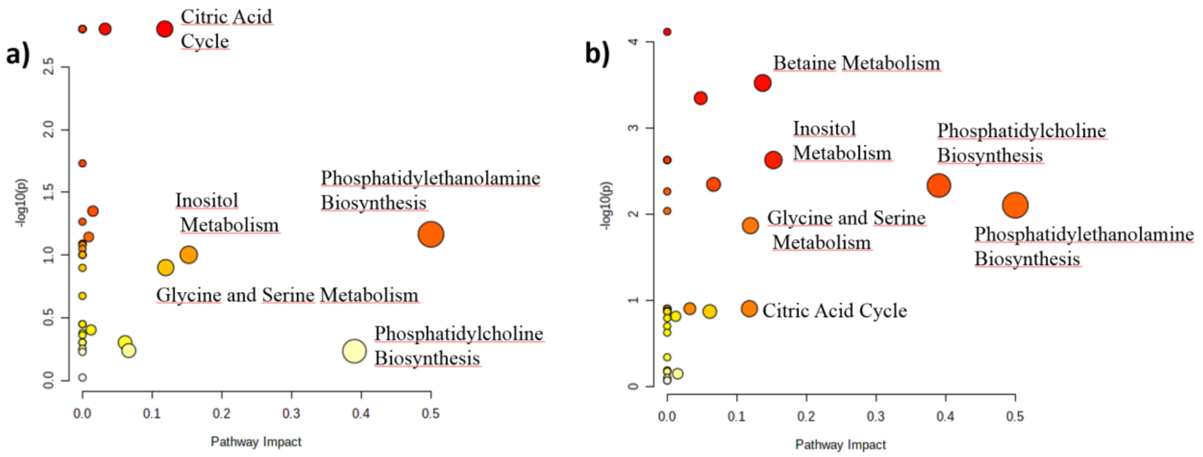

Figure S9. Metabolome views obtained from the analysis of the metabolic differences between G1 (stage 1) vs. G2 (stage 1) tumors (a) and between G2 (stage 2+3) vs. G3 (stage 2+3) tumors (b). The image was created using Metaboanalyst 4.0 software (https://www.metaboanalyst.ca).

6. Multivariate analysis of the merged aliphatic (0.8-4.8 ppm) and aromatic (5.2-8.4 ppm) regions of the HR MAS NMR spectra

Analysis of the contribution of the aromatic region (5.2-8.4 ppm) to the total area under the HR MAS NMR spectra

The free induction decay signals were multiplied by an exponential function (0.3 Hz), Fourier-transformed, phased and baseline corrected in Topspin 3.1 software (Bruker BioSpin GmbH). The spectra were referenced to formate peak (at 8.44 ppm) in Mestrenova software (Santiago de Compostela, Spain). The low (5.2-8.4 ppm) and high field (0.8-4.8 ppm) regions were integrated.

Table S12 shows the contribution of the aromatic region to the total area under the HR MAS NMR spectra (after excluding the residual water region) for the analyzed groups. Figure S10 presents the exemplary spectrum for which this contribution is equal to 2.3%.

Group	Contribution of the aromatic region to the total area under HR MAS NMR spectra<br>Median (25% – 75% percentiles)
Endometrial cancer, G1	1.4 (1.1-1.6) %
Endometrial cancer, G2	1.8 (1.5-2.2) %
Endometrial cancer, G3	2.3 (1.9-2.9) %
Control tissue	2.1 (1.9-2.2) %

Table S12. Contribution of the aromatic region (5.2-8.4 ppm) to the total area under the HR MAS NMR spectra (after excluding the residual water region).

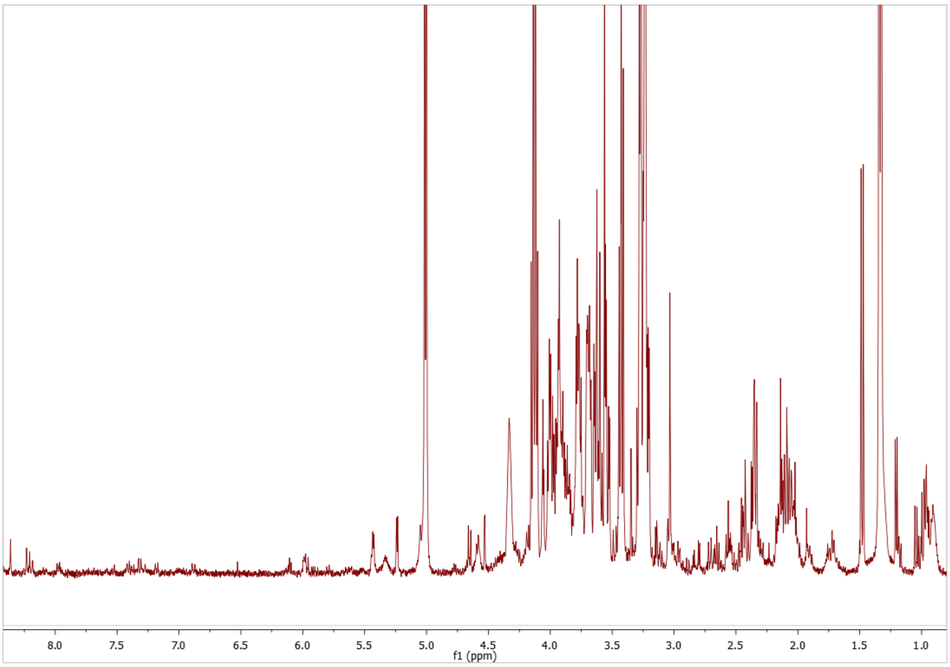

Figure S10. Exemplary HR MAS NMR spectrum (apodized with an exponential function of 0.3 Hz) obtained from endometrial cancer (G3). The contribution of the aromatic region to the total area under the spectrum is equal to 2.3%. The image was created in Mestrenova 10.0.2 software (www.metsrelab.com).

Normalization

Probabilistic quotient normalization (PQN) was used to make the spectra comparable to each other in our work [Dieterle, F., Ross, A., Schlotterbeck, G. & Senn, H. Probabilistic Quotient Normalization as Robust Method to Account for Dilution of Complex Biological Mixtures. Application in 1H NMR Metabonomics. *Analytical Chemistry* 78, 4281-4290 (2006)].

This method starts with the adjustment of the total intensity of each individual spectrum to the same value. Then, the ratios of the signal intensities between a given spectrum and a reference one (the median spectrum from the control group in our work) are calculated. The median of these ratios for a given spectrum is a normalization factor.

To avoid a negative impact of noise on this normalization factor value, the aromatic region was excluded from the normalization procedure. Taking a relatively small contribution of this region to the total area into account (Table S12), the exclusion of this part of the spectrum from the normalization procedure is not a critical step in our analysis. The obtained normalization factors for each spectrum were consistently used both for the aliphatic and aromatic regions.

Merging of aromatic and aliphatic regions into a single data matrix

The consistently normalized aliphatic (0.8 – 4.8 ppm, apodized with an exponential function 0.3 Hz) and aromatic (5.2 – 8.4 ppm, apodized with an exponential function 3 Hz) regions of the HR MAS NMR spectra were merged into a single data matrix. This matrix was centered and Pareto scaled before a multivariate analysis.

Multivariate analysis

The multivariate analysis of the merged aromatic and aliphatic regions was conducted according to the scheme presented in Figure S11. This scheme is similar to that presented in Figure 1 (the analysis of the aliphatic region, main manuscript file). The models computed from the merged aliphatic and aromatic regions are marked with a superscript ^{M} (PCA model 1^{M}, PLS-DA model 2^{M}, etc).

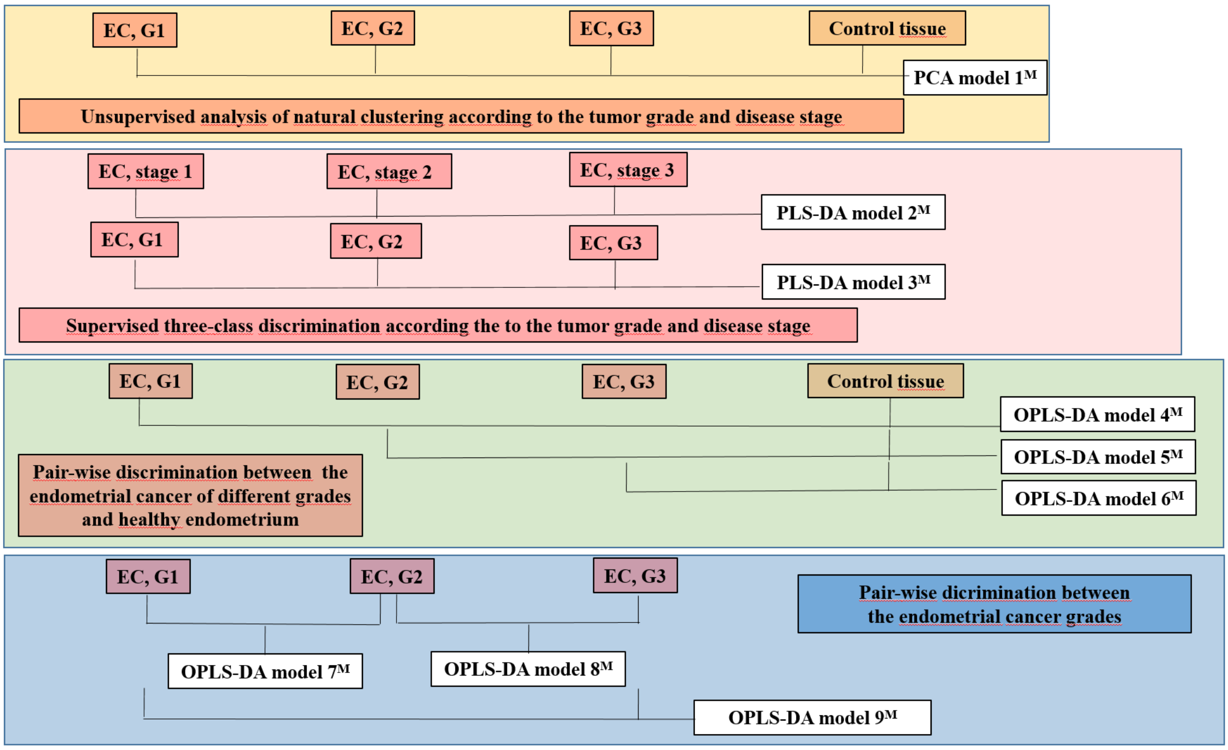

Figure S11. The scheme presenting the constructed multivariate models.

Results

Unsupervised analysis of natural clustering of patients according to the tumor grade and disease stage - Model PCA 1^M

The scores and loadings plots obtained from the model PCA 1^M are presented in Figure S12.

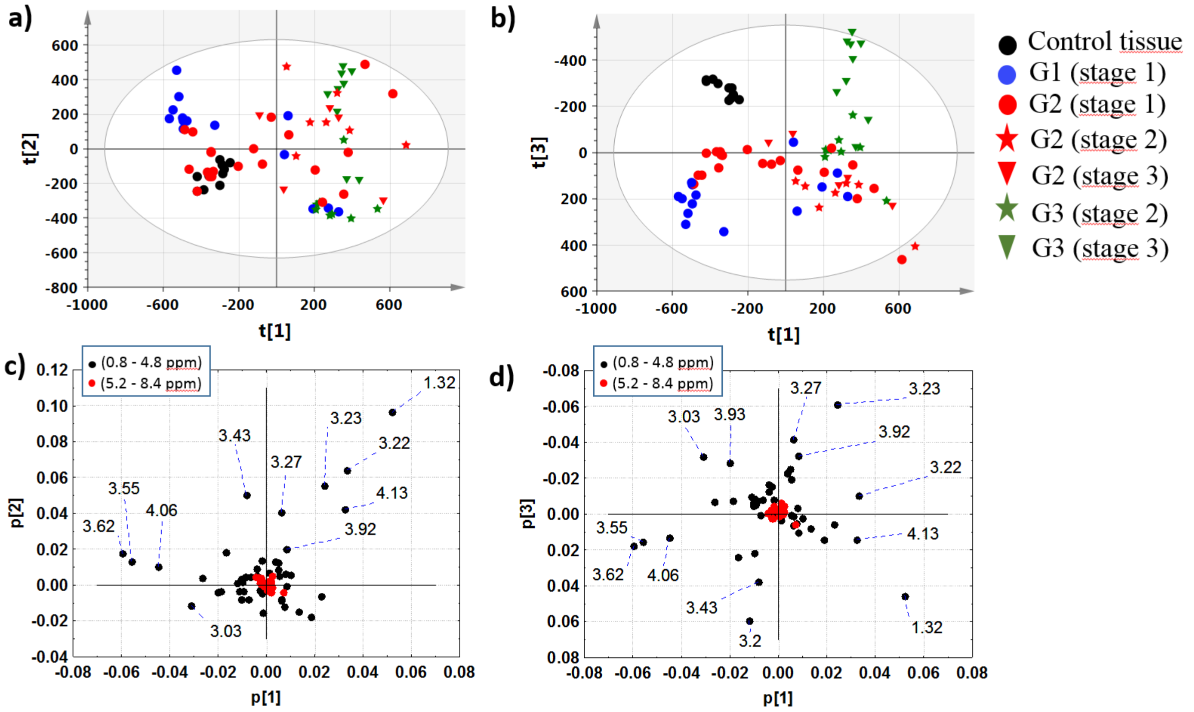

Figure S12. The PCA scores plots obtained from the model 1^M (in the parentheses there are the percentage values of the total variation in the given projection plane) for: (a) PC1-PC2 (43.2 %), (b) PC1-PC3 (34.5 %). The loadings plots for: (c) PC1-PC2 (d), PC1-PC3. The points in the scores plots are marked according to the disease stage and the tumors grade, while the points in the weights plots are labeled according to the chemical shifts. For clarity, the most important chemical shifts are presented. The scores plots (a-b) were created using SIMCA-P 15.0 software package (https://www.sartorius.com). The loadings plots (c-d) were created using Statistica 12.5 software (www.statsoft.pl) based on data exported from SIMCA-P 15.0 software package (https://www.sartorius.com).

Supervised three-class discrimination according the to the tumor grade and the disease stage (Models PLS-DA 2^M and PLS-DA 3^M)

The cross-validated scores and weights plots obtained from the PLS-DA models 2^M and 3^M are presented in Figure S13.

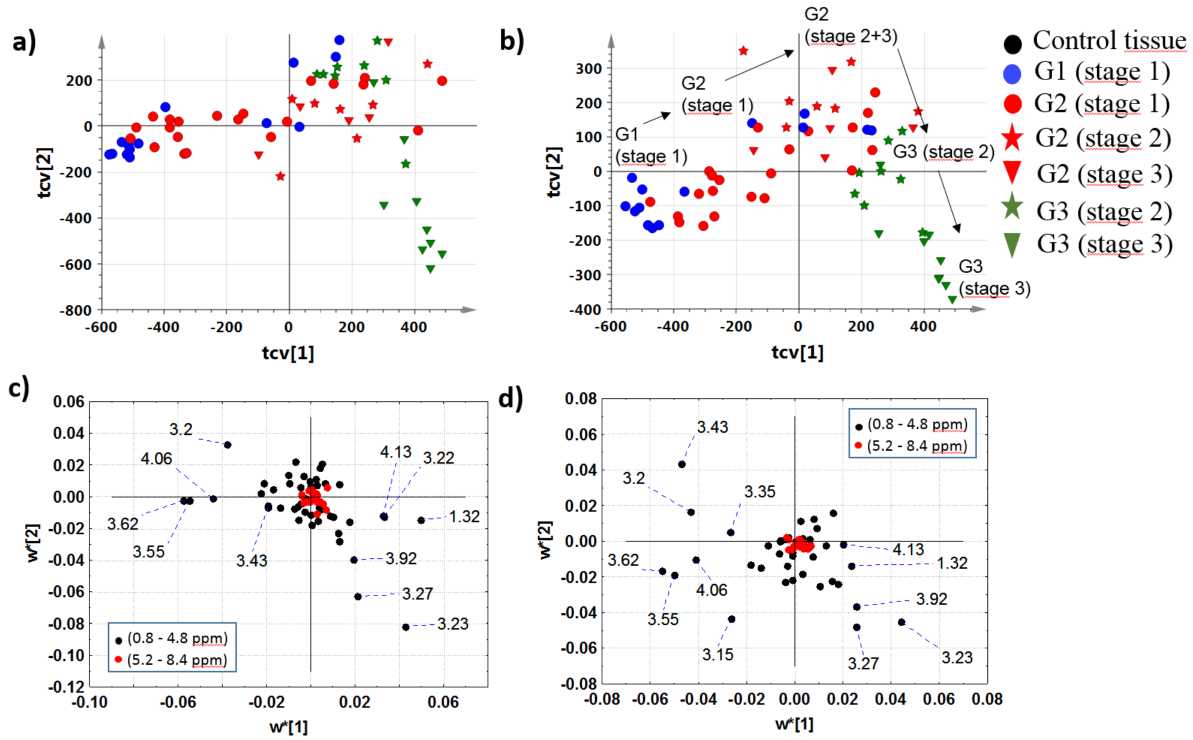

Figure S13. The cross-validated PLS-DA scores (a) and weights (c) plots obtained from the model 2^M differentiating the patients according to the disease stage (2 components, R2X = 42 %, R2Y = 46.1 %, Q2 = 37.6 %). The cross-validated PLS-DA scores (b) and weights (d) plots obtained from the model 3^M differentiating the patients according to the tumor grade (3 components, R2X = 43.2 , R2Y = 69.5 %, Q2 = 40.5 %). The points in the scores plots are marked according to the disease stage and the tumors grade, while the points in the weights plots are labeled according to the chemical shifts. For clarity, the most important chemical shifts are presented. The scores plots (a-b) were created using SIMCA-P 15.0 software package (https://www.sartorius.com). The loadings plots (c-d) were created using Statistica 12.5 software (www.statsoft.pl) based on data exported from SIMCA-P 15.0 software package (https://www.sartorius.com).

It is apparent that the patterns visible in the scores plots obtained from the models PCA 1^M, PLS-DA 2^M and PLS-DA 3^M (Figures S12 and S13) are similar to those obtained from the models PCA 1, PLS-DA 2 and PLS-DA 3 (Figures 3 and 4, main manuscript file). The presented loadings plots indicate a minor contribution of the aromatic region to the principal components.

Pair-wise discrimination between the endometrial cancer of different grades and healthy endometrium (OPLS-DA model 4^M, OPLS-DA model 5^M, OPLS-DA model 6^M)

The scores and loadings plots obtained from the OPLS-DA models 4^M, 5^M and 6^M are presented in Figure S14. The p(corr)[1] vs VIP plots for the aromatic range are also shown in this plot. The number of the model components, the fractions of the X and Y variation explained by the predictive and orthogonal components (R2X and R2Y), the fractions of the Y variation predicted by the models (Q2), the p-values from the CV-Anova test and the results from the permutation testing are presented in Table S13.

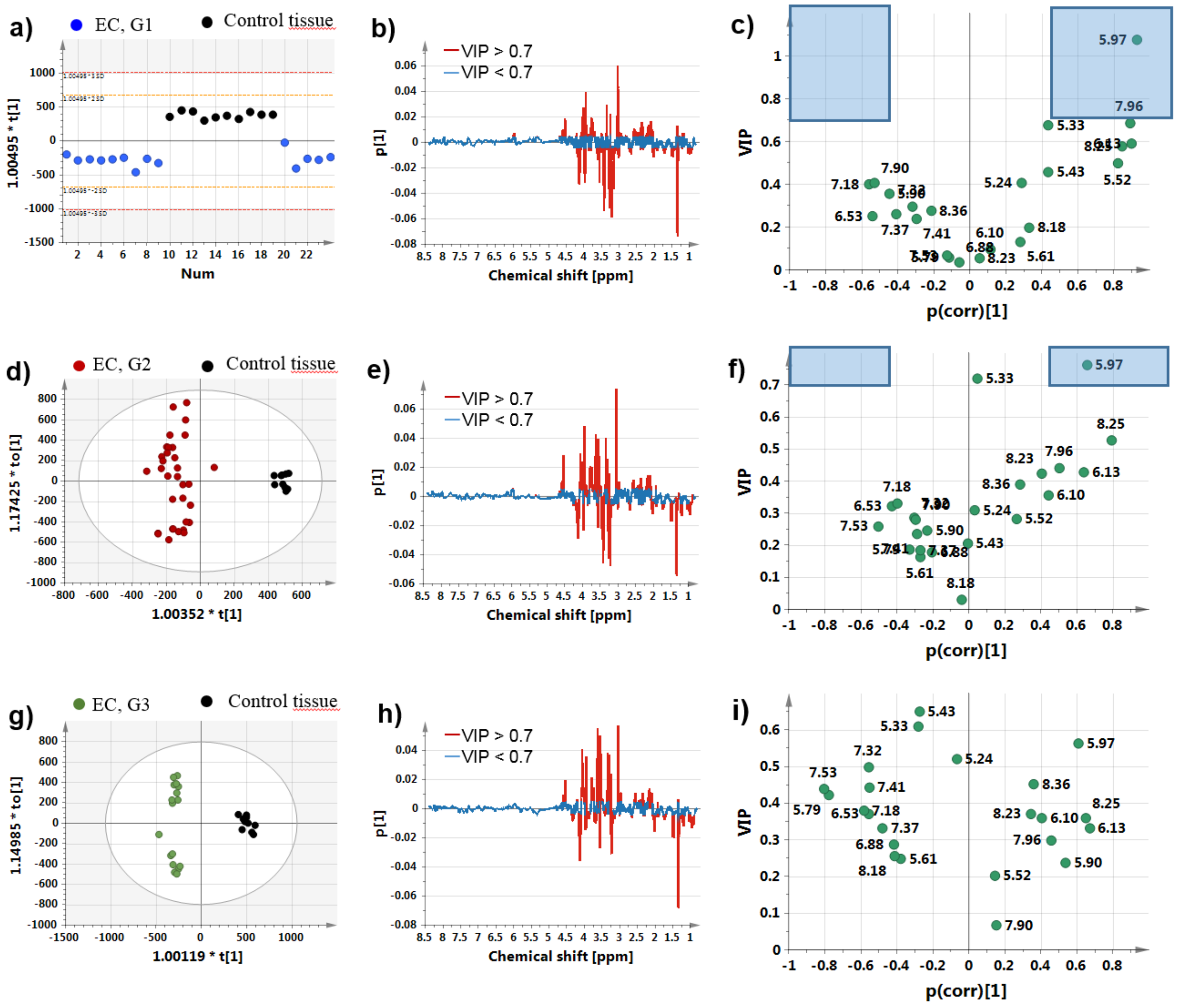

Figure S14. The OPLS-DA models: (a) the scores plot (b) the loadings plot and (c) p(corr)[1] vs VIP plot for the aromatic region obtained from the model 4^M; (d) the scores plot (e) the loadings plot and (f) p(corr)[1] vs VIP plot for the aromatic region obtained from the model 5^M; (g) the scores plot (h) the loadings plot and (i) p(corr)[1] vs VIP plot for the aromatic region obtained from the model 6^M. Loadings are colored according to VIP values (b), (e), (h). The sections characterized by |p(corr)[1]| > 0.45 and VIP > 0.7 are marked in blue in the p(corr)[1] vs VIP plots (c), (f), (i). The image was created using SIMCA-P 15.0 software package (https://www.sartorius.com).

Model	CV-Anova p-value	Number of components	R2X [%]	R2Y [%]	Q2 [%]	Intercepts obtained from the permutation test	
						R2Y [%]	Q2Y[%]
G1 vs Control tissue (model 4^M)	1.50179e-12	1 predictive	31.6	94.5	92.5	7.1	-42.6
		0 orthogonal	-				
G2 vs Control tissue (model 5^M)	1.73815e-20	1 predictive	18.2	94.4	92.2	25	-38.8
		1 orthogonal	28.7				
G3 vs Control tissue (model 6^M)	8.81715e-18	1 predictive	38.3	98.3	97.7	28.3	-45.9
		1 orthogonal	20.4				

Table S13. OPLS-DA models (4^M-6^M) diagnostics. Number of the OPLS-DA model components, the p-values obtained from the CV Anova test, the fractions of the total X and Y variation explained by the model (R2X, R2Y), the fractions of the total Y variation that can be predicted by the model (Q2), the intercepts values obtained from the permutation tests representing the values of R2Y and Q2 of the purely random models.

Pair-wise discrimination between different grades of endometrial cancer (OPLS-DA model 7^M, OPLS-DA model 8^M, OPLS-DA model 9^M)

The scores and loadings plots obtained from the OPLS-DA models 7^M, 8^M and 9^M are presented in Figure S15. The p(corr)[1] vs VIP plots for the aromatic range are also shown in this plot. The number of the model components, the fractions of the X and Y variation explained by the predictive and orthogonal components (R2X and R2Y), the fractions of the Y variation predicted by the models (Q2), the p-values from the CV-Anova test and the results from the permutation testing are presented in Table S14.

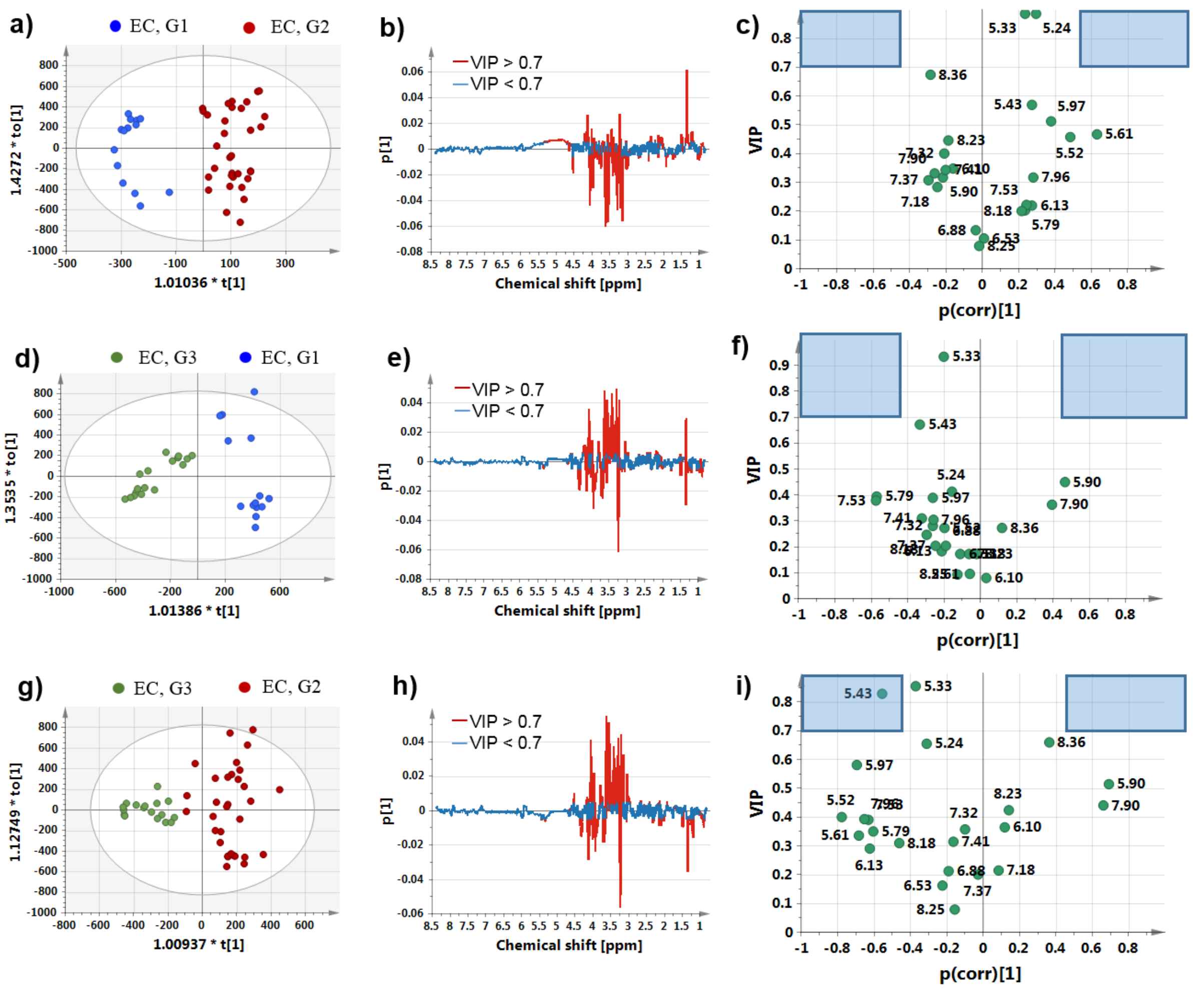

Figure S15. The OPLS-DA models: (a) the scores plot (b) the loadings plot and (c) p(corr)[1] vs VIP plot for the aromatic region obtained from the model 7^{M}; (d) the scores plot (e) the loadings plot and (f) p(corr)[1] vs VIP plot for the aromatic region obtained from the model 9^{M}; (g) the scores plot (h) the loadings plot and (i) p(corr)[1] vs VIP plot for the aromatic region obtained from the model 8^{M}. Loadings are colored according to VIP values (b), (e), (h). The sections characterized by |p(corr)[1]| > 0.45 and VIP > 0.7 are marked in blue in the p(corr)[1] vs VIP plots (c), (f), (i). The image was created using SIMCA-P 15.0 software package (https://www.sartorius.com).

Model	CV-Anova p-value	Number of components	R2X [%]	R2Y [%]	Q2 [%]	Intercepts obtained from the permutation test	
						R2Y [%]	Q2Y[%]
G1 vs G2 (model 7^M)	5.19327e-05	1 predictive	7.83	87.8	56.0	56.0	-60.0
		3 orthogonal	50.4				
G2 vs G3 (model 8^M)	5.87298e-12	1 predictive	14.4	81.8	72.1	28.3	-38.3
		1 orthogonal	23.2				
G1 vs G3 (model 9^M)	1.62499e-08	1 predictive	30.1	85.8	79.1	28.8	-47.1
		1 orthogonal	22.1				

Table S14. OPLS-DA models (7^M-9^M) diagnostics. Number of the OPLS-DA model components, the p-values obtained from the CV Anova test, the fractions of the total X and Y variation explained by the model (R2X, R2Y), the fractions of the total Y variation that can be predicted by the model (Q2), the intercepts values obtained from the permutation tests representing the values of R2Y and Q2 of the purely random models.

The results presented in Figures S14 and S15 indicate that the latent variables obtained from the OPLS-DA models 4^M -9^M are characterized by a minor contribution from the aromatic region. The $|p(corr)[1]| > 0.45$ and $VIP > 0.7$ requirement is fulfilled only for the signal at 5.97 ppm (UDP - sugar) in the OPLS-DA models 4^M and 5^M and for the signal at 5.43 ppm (glycogen) in the OPLS-DA model 9^M .

Therefore, separate multivariate analysis was performed for the aromatic region.

7. Multivariate analysis of the spectral region from 5.2 to 8.4 ppm

Preprocessing

The free induction decay signals were multiplied by an exponential function (3 Hz), Fourier-transformed, phased, baseline corrected and referenced to formate peak at 8.44 ppm. The data were analyzed at full resolution. For consistency of the results the region from 5.2 to 8.4 ppm was normalized using the scaling factors obtained from probabilistic quotient normalization of the region from 0.8 to 4.8 ppm.

Multivariate analysis

The pre-processed data were imported to SIMCA-P 15.0 software (Umetrics, Sweden) for multivariate modeling. Before the modeling, the data were mean centered and Pareto scaled. The scheme of the analyses is presented in Figure S16.

Principal Component Analysis (PCA) was used to obtain the initial information about the natural grouping of the spectra acquired from the cancer samples (according to the tumor grade and the disease stage) and the healthy tissue (model S5). The clustering of tumors according to grade and stage was also examined using Partial Least-Squares Discriminant Analysis (PLS-DA) (models S6 and S7).

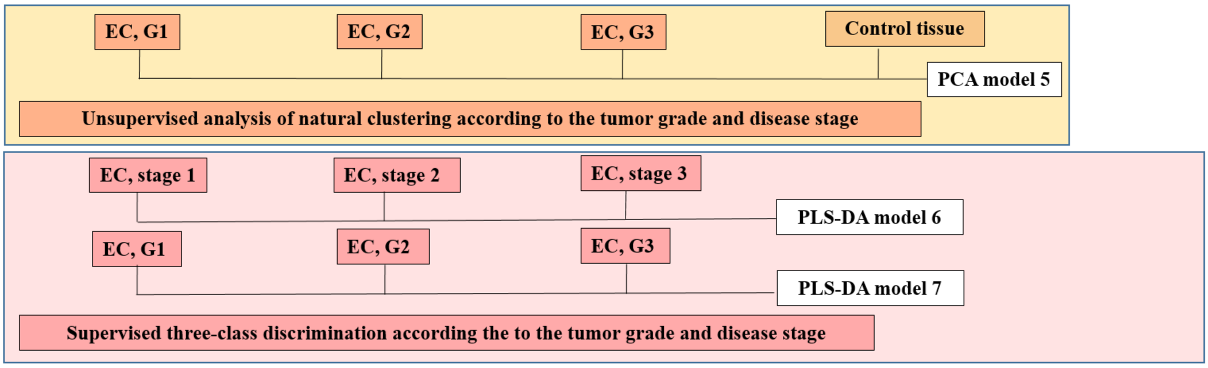

Figure S16. The scheme presenting the constructed multivariate models.

Results

Figure S17 presents the average CPMG spectra (region from 5.2 to 8.4 ppm) acquired from the different grades of endometrial cancer and the control tissue. The tentatively assigned metabolites are marked in this figure and listed in Table S15.

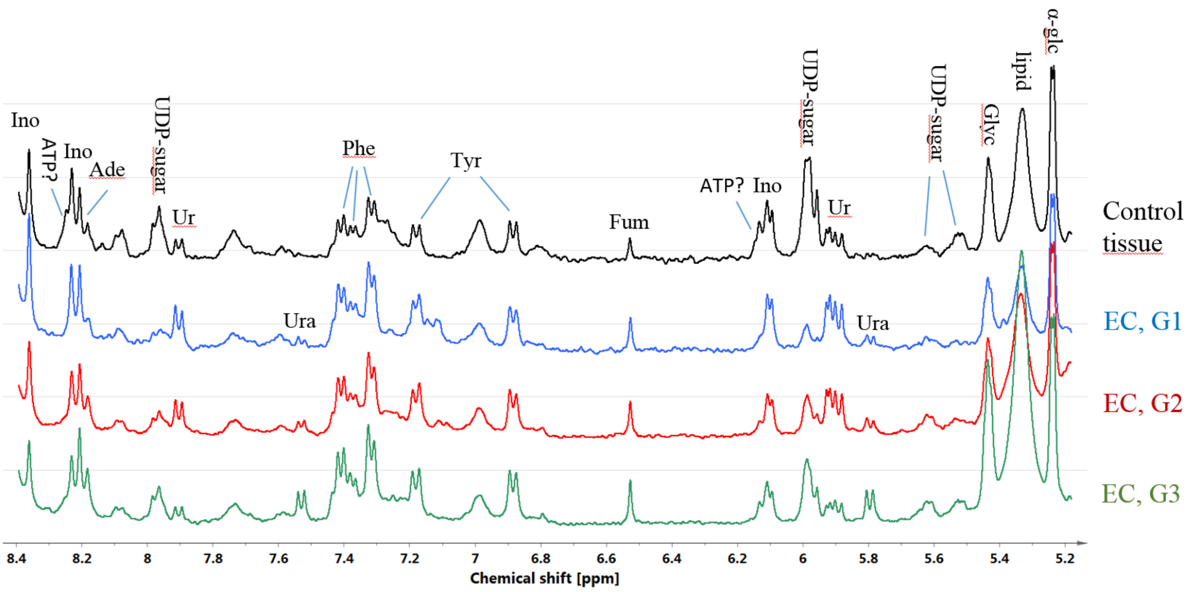

Figure S17. Average ^{1}H HR-MAS NMR CPMG spectra obtained from the G1, G2 and G3 endometrial tumors and the control tissue. The image was created using SIMCA-P 15.0 software package (https://www.sartorius.com). α-glc – α-glucose, Lipid – unsaturated lipids, Glyc – glycogen, Ur – uridine, UDP sugar - Uridine diphosphate sugar, Ura – uracil, Ino – Inosine, ATP - adenosine triphosphate, Fum – fumarate, Tyr – tyrosine, Phe – phenylalanine, Ade - adenine

**Metabolite**	**Chemical shift**
α-glucose	5.24 ppm
Unsaturated lipids	5.33 ppm
Glycogen	5.43 ppm
Uracil	5.79 ppm, 7.53 ppm
UDP-sugars	5.52 ppm, 5.62 ppm, 5.97 ppm, 7.96 ppm
Uridine	5.90 ppm, 7.90 ppm
Fumarate	6.53 ppm
Tyrosine	6.88 ppm, 7.18 ppm
Phenylalanine	7.32 ppm, 7.37 ppm, 7.41 ppm
Inosine	6.1 ppm, 8.23 ppm, 8.36 ppm
Adenine	8.18 ppm, 8.20 ppm
ATP?	6.13 ppm, 8.25 ppm

Table S15. The chemical shift assignments in the spectral region from 5.2 to 8.4 ppm.

PCA model S5

Results obtained from PCA model S5 are presented in Figure S18. The control tissue was found to be separated from tumors of all grades in the PC1-PC3 projection plane (Figure S18a). The high levels of UDP-sugars (5.97 ppm, 7.96 ppm, 6.1 ppm), ATP (8.25 ppm, 6.13 ppm) and inosine (8.23 ppm and 6.10 ppm) in the non-transformed endometrium are mainly responsible for this separation (Figure S18c). Although there is a substantial overlap between the different tumor grades and disease stages in the PC2-PC3 projection plane, the PC2 scores tend to separate G1 (stage 1) and G2 (stage 1) from the G2 (stage 2+3) and G3 (stage 2+3) tumors (Figure 18b). This separation is mainly caused by the lower phenylalanine (7.41 ppm, 7.32 ppm, 7.37 ppm), uracil (5.79 ppm, 7.53 ppm) and the higher inosine (8.23 ppm, 8.36 ppm, 6.10 ppm) and uridine (7.90 ppm and 5.90 ppm) in G1 (stage 1) and G2 (stage 1) tumors than in G2 (stage 2+3) and G3 (stage 2+3) tumors (Figure 18d).

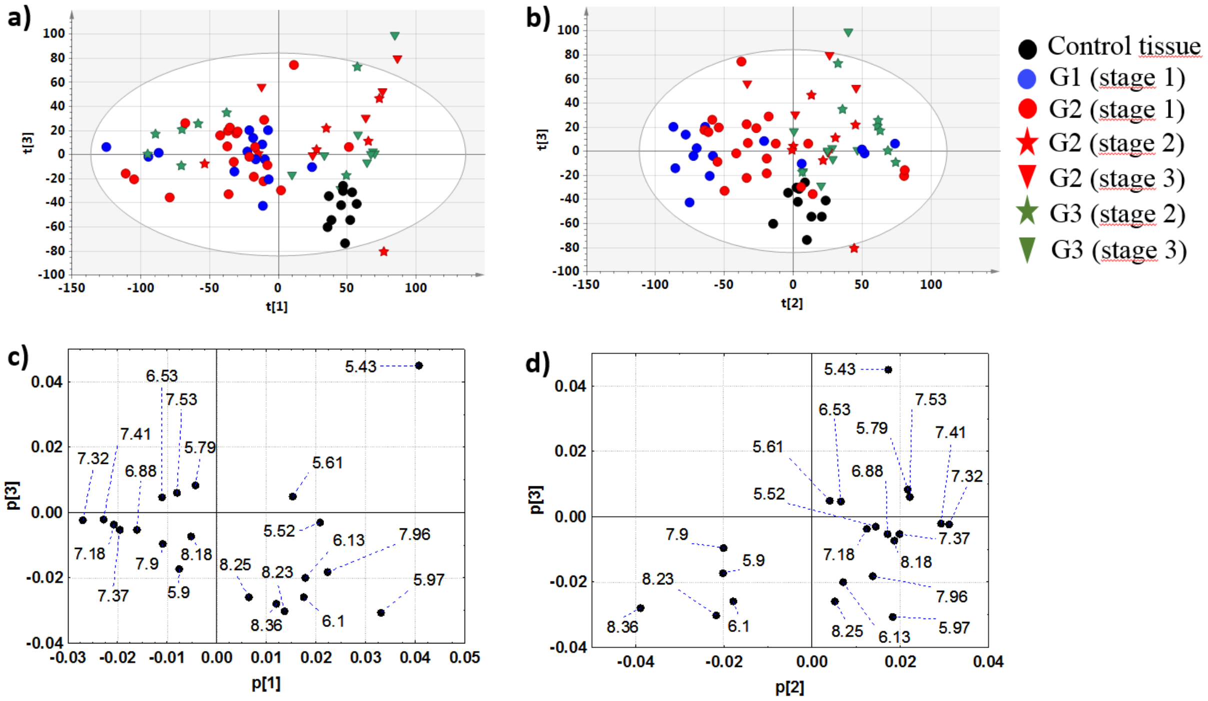

Figure S18. The PCA scores plots obtained from the model S5 (in the parentheses there are the percentage values of the total variation in the given projection plane) for: (a) PC1-PC31 (28.2%), (b) PC2-PC3 (21.5%). The loadings plots for: (a) PC1-PC3 (c), PC2-PC3. The points in the scores plots are marked according to the disease stage and tumors grade, while the points in the weights plots are labeled according to the chemical shifts. For clarity, the most important chemical shifts are presented. The scores plots (a-b) were created using SIMCA-P 15.0 software package (https://www.sartorius.com). The loadings plots (c-d) were created using Statistica 12.5 software (www.statsoft.pl) based on data exported from SIMCA-P 15.0 software package (https://www.sartorius.com).

PLS-DA models S6 and S7

Table S16 shows the number of the components, the fractions of the X and Y variation explained by the PLS-DA models S6 (differentiating the patients according to the disease stage) and S7 (differentiating the tumors according to the grade), the fractions of the Y variation predicted by the models and p value obtained from CV-Anova test.

Model	CV-Anova p-value	Number of components	R2X [%]	R2Y [%]	Q2 [%]
Stage 1 vs. Stage 2 vs. Stage 3 (model S6)	1.30414e-13	3	38.4	65.7	45.5
G1 vs. G2 vs. G3 (model S7)	0.00786581	2	25.6	43.1	8.0

Table S16. PLS-DA models (S6 and S7) diagnostics. The number of the components, the fractions of the X and Y variation explained by the models (R2X and R2Y), the fractions of the Y variation predicted by the models (Q2) and p values obtained from CV-Anova test.

The cross-validated scores and loadings plots obtained from the PLS-DA models differentiating different disease stages (model S6) and tumor grades (model S7) are presented in Figure S19.

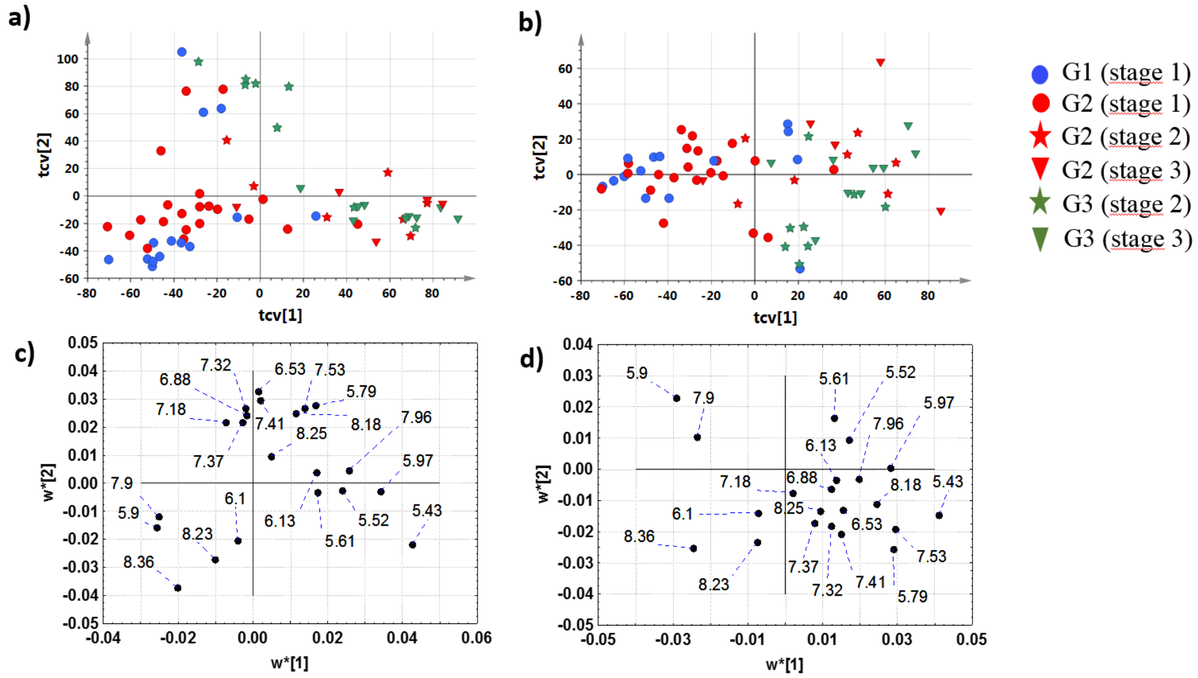

Figure S19. The cross-validated PLS-DA scores (a) and weights (c) plots obtained from the model S6 differentiating the patients according to the disease stage. The cross-validated PLS-DA scores (b) and weights (d) plots obtained from the model S7 differentiating the patients according to the tumor grade. The points in the scores plots are marked according to the disease stage and tumors grade, while the points in the weights plots are labeled according to the chemical shifts. For clarity, the most important chemical shifts are presented. The scores plots (a-b) were created using SIMCA-P 15.0 software package (https://www.sartorius.com). The weights plots (c-d) were created using Statistica 12.5 software (www.statsoft.pl) based on data exported from SIMCA-P 15.0 software package (https://www.sartorius.com).

Although G1(stage 1) and G2 (stage 1) tumors are separated from G2 (stage 2+3) and G3 (stage 2+3) in the scores plots obtained from both models (Figure S19a-b), the parameters presented in Table S16 indicate that the model differentiating the patients according to the disease stage (model S6) has a better performance than the model differentiating the tumors according to the

grade (model S7). The weights plot indicates that G2 (stage 2+3) and G3 (stage 2+3) tumors are characterized by the higher level of glycogen (5.43 ppm), UDP sugars (5.97 ppm, 7.96 ppm, 5.52 ppm, 5.61 ppm) and lower level of uridine (5.9 ppm, 7.9 ppm) and inosine (8.36 ppm, 8.23 ppm) than G1 (stage 1) and G2 (stage 2) tumors (Figure S19c).
